# Captodative Radicals Enable the Coexistence of Monomer and Dimer Single-Molecule Junctions with 100-Fold Difference in Conductance

**DOI:** 10.1021/jacs.6c01727

**Published:** 2026-06-09

**Authors:** Weiyi Guo, Shuai Yao, Xueling Xu, Taotao Lu, Chaochao Xie, Yaxuan Zhang, Jiahao Wang, Hongliang Chen, Xuefeng Tan, Yangyang Shen, Haixing Li

**Affiliations:** † Department of Physics, 53025 City University of Hong Kong, Kowloon 999077, Hong Kong SAR, China; ‡ Frontier Institute of Science and Technology, 12480 Xi’an Jiaotong University, Yanxiang Road 99, Xi’an 710045, China; § Department of Chemistry, City University of Hong Kong, Kowloon 999077, Hong Kong SAR, China; ⊥ Department of Chemistry, Stoddart Institute of Molecular Science, and ZJU-Hangzhou Global Scientific and Technological Innovation Center, 12377 Zhejiang University, Hangzhou 310027, China

## Abstract

Atomic-scale manipulation of chemical bond cleavage and formation offers significant advantages of extending the reaction controllability to the single-molecule regime and enabling the elucidation of fundamental reaction mechanisms. Herein, we drive reactions between two chemical species, open-shell monomeric radicals and closed-shell dimers in single-molecule junctions. Both monomers and dimers form a C−Au covalently linked highly conducting junction in situ, with the conductance being about 100× higher than that of the dimer junction. First-principles calculations suggest that the substitution groups on the carbon in the C­(sp^3^)−Au linkage bond effectively tunes the molecular junction conductance. Notably, captodative radicals enable the formation of weakly bonded spin–spin interactions in solution, yielding diamagnetic dimers, and we can reversibly switch between the dimer and the weakly bonded radical pair by mechanically controlling the gap between the two electrodes.

## Graphical abstract

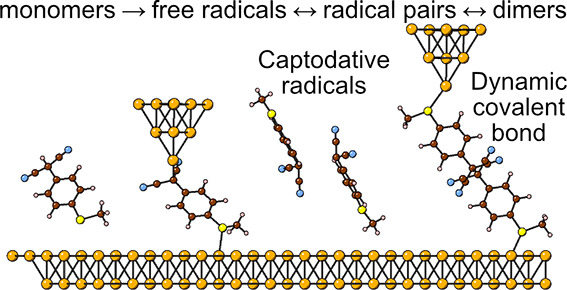

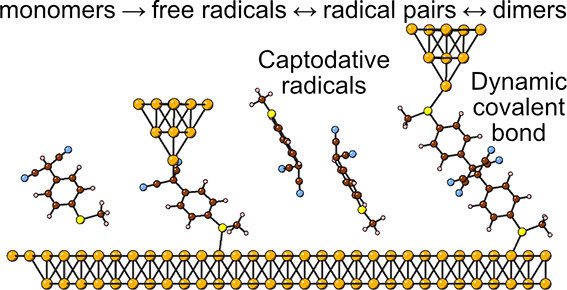

## Introduction

Radical-based single-molecule junctions, with their unique open-shell structures, have been used for investigating charge transport,^1–3^ thermoelectric properties,^4^ the Kondo effect,^5,6^ and magnetoresistance.^7,8^ Captodative radicals, however, have rarely been used as active components for constructing single-molecule devices. Captodative (or push–pull) radicals are a class of radicals that are substituted with both electron-donating, such as amino (−NR_2_) or alkoxy (−OR), and electron-withdrawing, such as cyano (−CN) or carbonyl (−RC=O), groups, exhibiting an enhanced stability due to a delocalization of the unpaired electron.^9–12^ In particular, aryl dicyanomethyl radicals are thermally stable and are not air-sensitive,^13^ and given the inherent reactivity of their radical centers, they are promising building blocks for constructing dynamic covalent assemblies^14,15^ and nanoscale electronic devices. On the other hand, the open-shell captodative radicals can undergo self-association to form weakly bound closed-shell homodimers.^16,17^ This reversible radical-dimer equilibrium enables diverse applications of captodative radicals in areas including spintronics,^18,19^ single spin-crossover molecules,^20–22^ and structurally dynamic materials^23,24^ but not yet in single-molecule electronics.

Undercoordinated Au atoms, although usually forming dative interactions with organic anchoring groups, are quite reactive and can also play an active role in driving reactions that occur in break-junction experiments.^25–28^ In single radical junctions, the role of Au atoms can go beyond the formation of organic-metal interactions, when more than one type of junction is formed and multiple chemical species reach a reaction equilibrium on the Au surface. The catalytic effect of undercoordinated Au atoms,^29^ especially on the reactive radical species, needs to be considered.

In this study, we demonstrate the coexistence of monomer and dimer single-molecule junctions by using the captodative aryl dicyanomethyl radicals under electric fields with Au electrodes. Specifically, scanning tunneling microscope-based break junction (STM-BJ) experiments of both the monomer **1** and the dimer **2** show two conductance peaks labeled as highG and lowG. LowG is from a dimer junction, and through measurements of series of control molecules, we confirm that highG is from C−Au covalently linked junction formed by the monomeric radical. The conductance properties of C­(sp^3^)−Au linked junctions are further corroborated and analyzed by first-principles calculations. Our results show that the reversible interconversion between monomeric radicals and dimers leads to the formation of two distinct junctions, and by inhibiting the dissociation of radical pairs into free radicals, we can primarily form only lowG junctions. Moreover, utilizing the sub-Ångström level of control of the STM-BJ technique, we mechanically induce transitions between a radical pair and a dimer through modulations of the distance between two electrodes. We finally extend the dehydrogenative dimerization of the dicyanomethyl group to phenethyl systems, achieving products that would otherwise be challenging to obtain through traditional synthetic approaches.

## Results and Discussion

We first synthesize and examine molecule **1** (chemical structure in Figure 1a), a benzene structure terminated with a thiomethyl group on one end and a dicyanomethyl group on the other, which could be easily accessed by reacting 4-bromothioanisole and malononitrile under palladium catalysis (details are given in Supporting Information Sections I and IV). In **1**, the aryl dicyanomethyl group undergoes dehydrogenation to generate the captodative aryl dicyanomethyl radical (**1R**), and such a radical is stabilized by the neighboring electron-withdrawing (captor, −CN group) and electron-releasing (donor, phenyl group) substituents on a radical center. Within the resonance model shown in Figure 1a, two resonance structures contribute to the overall resonance hybrid. It has been demonstrated that such a system maintains an equilibrium between captodative radicals (**1R**) and the corresponding diamagnetic radical dimer (**1R** pair), and the radical dimer undergoes a subsequent head-to-head dimerization to form compound **2** (illustrated in Figure 1b). This step completes the two-stage transformation from the initial aryl dicyanomethyl precursor (**1**) to the final coupled product (**2**) through a radical-mediated pathway. We indeed find that the dimerization of **1** into forming **2** proceeds spontaneously in solution in air (Figure S1 in the Supporting Information), and different reaction rate constants are seen for the two solvents hexane and isopropanol used (Tables S1 and S2 in the Supporting Information).

**Figure 1:**
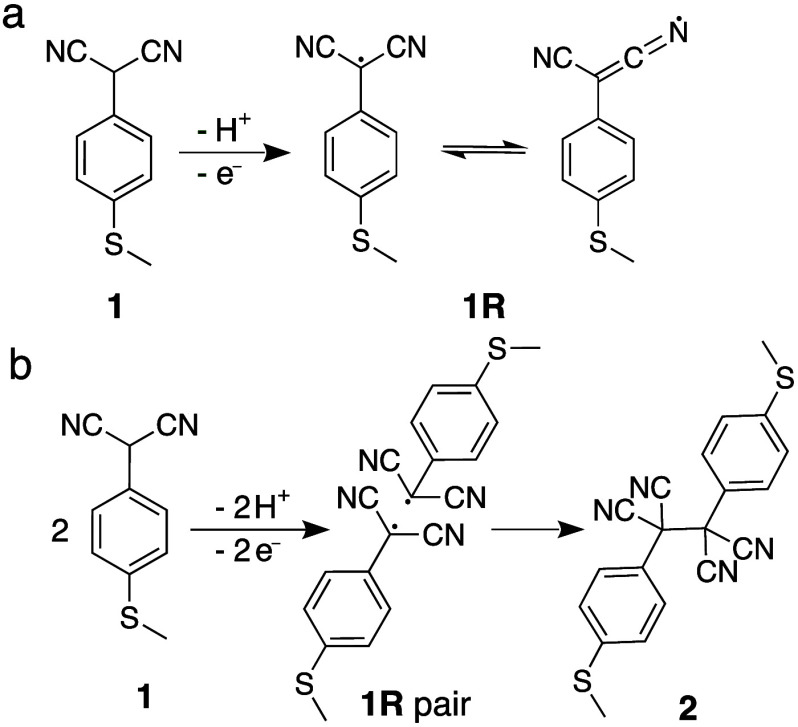
Reaction of **1** in forming radical **1R** and dimer **2**. (a) Formation of radical **1R** from **1**. (b) Formation of radical pair from **1**, and homocoupling reaction of **1R** pair in forming compound **2**.

We subject 1 mM **1** in 1,2,4-trichlorobenzene (TCB) solution under an applied electric field provided by the STM-BJ technique^30,31^ and monitor the single-molecule conductance of **1**. Experimental details are provided in the Methods section. Briefly, in this technique, a gold STM tip is repeatedly brought in and out of contact with a gold-coated substrate. Conductance (current/voltage) is recorded as a function of the relative tip/substrate displacement while the tip is retracted. Conductance traces exhibit 1G_0_ (G_0_ = 2e^2^/*h*) plateau, which corresponds to the single Au–Au atomic contact, and additional plateaus below 1G_0_, which only appear when molecular solution is added. These plateaus below 1G_0_ are attributed to the Au–molecule–Au junctions. Data without selection are used to construct logarithmically binned one-dimensional (1D) and two-dimensional (2D) conductance histograms. As shown in Figure 2g and 2h, in experiment of **1** performed under 540 mV, we observe two conductance peaks: one peak at 4.6 × 10^–3^ G_0_ with a molecular elongation length of ∼0.25 nm, labeled as 1highG, and a second peak located at 4.0 × 10^–5^ G_0_ with a molecular elongation length of ∼0.67 nm, termed as 1lowG.

**Figure 2:**
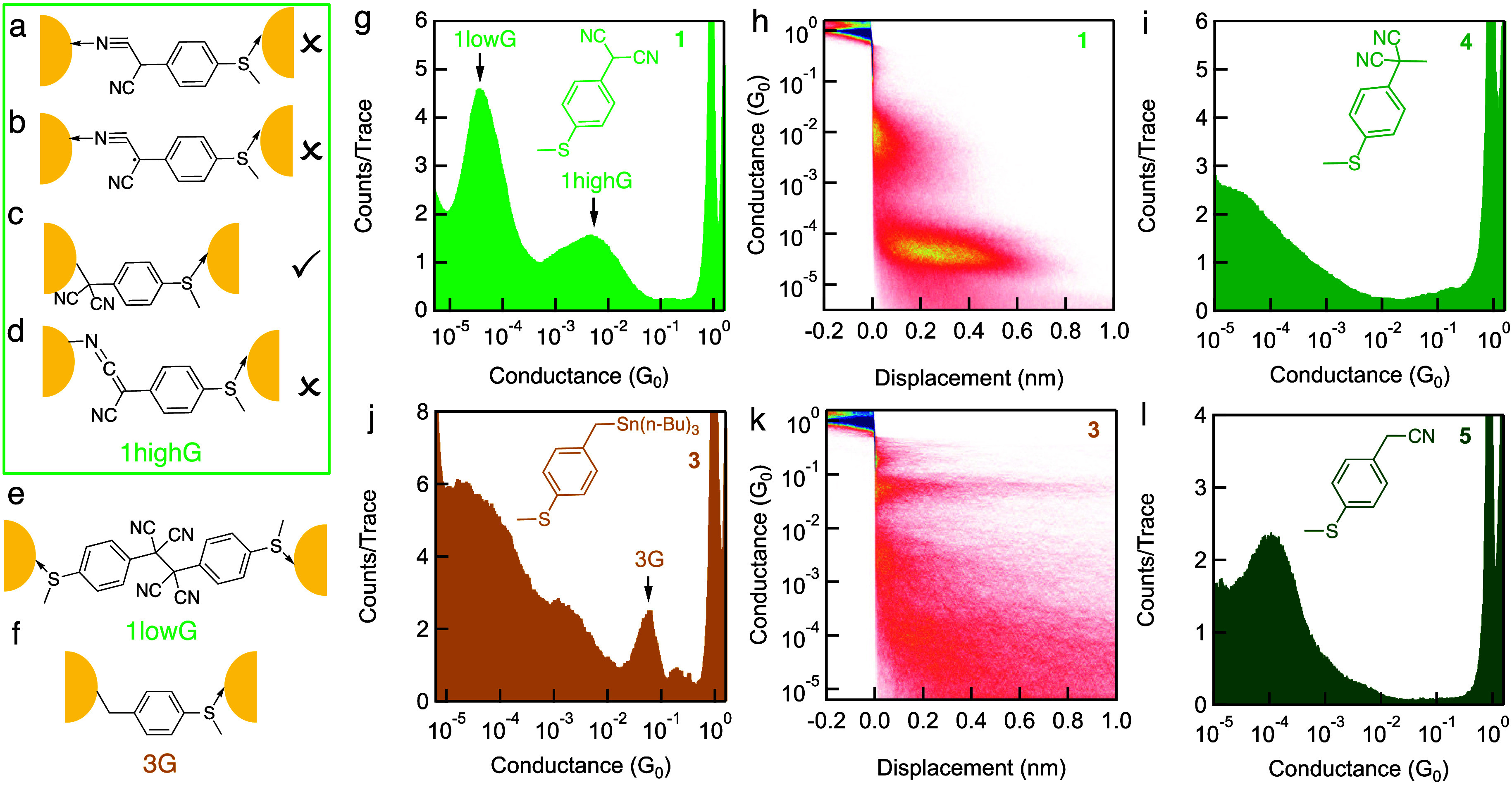
Break junction measurements of **1** in forming **1** radical and dimer **2** junctions. Possible binding schemes for (a) **1** and (b–d) **1R** assigned for 1highG in STM-BJ experiments of **1**. (e) Schematic illustration of molecular junctions assigned for 1lowG in STM-BJ experiments of **1**. (f) Schematic illustration of molecular junctions assigned for 3G in STM-BJ experiments of **3**. (g) 1D and (h) 2D histograms of **1** measured in TCB under 540 mV. (i) 1D histograms of **4** measured in TCB under 540 mV. (j) 1D and (k) 2D histograms of **3** measured in TCB under 540 mV. (l) 1D histogram of **5** measured in TCB under 540 mV. Chemical structures of **1**, **3**, **4**, and **5** are shown as insets.

We first focus on 1highG. We propose that two chemical species, **1** or **1R**, are both possible to give rise to this conductance peak. In junctions formed by either **1** or **1R**, the thiomethyl linker group binds to the Au electrode on one side. For junction of **1**, cyano (−CN) group(s) could serve as the other anchoring group through a N→Au dative interaction (Figure 2a).^32^ To test this possibility, we compare the conductance properties of 1highG with those of compounds **4** and **5** (chemical structures in Figure 2i and 2l). We install a methyl group (−CH_3_) at the benzyl position in **1** for synthesizing **4** and also study **5** in which only one −CN terminal group is present. We do not observe a conductance peak for **4** (Figure 2i), possibly due to the lack of stable access to the −CN group(s) from the Au electrode, and find a conductance peak at 1.3 × 10^–4^ G_0_ with a molecular elongation length of ∼0.31 nm for **5** (Figure 2l and Figure S2), which is likely of a junction of **5** bound through SMe→Au and CN→Au dative bonds on the two ends. The lower conductance (×0.03) and larger junction elongation length for **5**, in comparison to 1highG, strongly indicates that 1highG does not result from the junction of **1**. Next, we consider all possible junctions formed by **1R**.

We propose three possible junctions formed from **1R**: (i) **1R** is bound to the gold electrodes through a CN→Au dative bond (Figure 2b), (ii) the carbon radical of **1R** is attached to gold electrodes through C–Au covalent bond (Figure 2c), and (iii) the nitrogen radical formed via resonance is bound to gold electrodes through N–Au covalent bond (Figure 2d). We suggest that, although it is extremely difficult to characterize the chemical nature of this linkage in situ, a major distinction should appear in their junction lengths: (i) and (iii) are expected to display a larger junction length than (ii), as they both contain two additional bonds in the junction in comparison to (ii). In order to evaluate the junction elongation length for scenario (ii), we design and synthesize the organotin compound **3** (chemical structure in Figure 2j). It has been demonstrated that the cleavage of trimethyltin or *n*-butyl-substituted tin termination group allows for the formation of a direct C–Au covalent contact,^27,33,34^ thus we assign the conductance peak at 6.3 × 10^–2^ G_0_ (Figure 2j and 2k, and Figure S3) to be the conductance for a C−Au linked junction 3G, as illustrated in Figure 2f, agreeing with the previously measured conductance values of similar compounds.^34–38^

We find that 1highG demonstrates an even shorter junction elongation length (∼0.25 nm) compared to that of 3G (∼0.29 nm), indicating that scenarios of (i) and (iii) are not possible, leaving us with (ii) being the only possible junction for 1highG, as depicted in Figure 2c. The attribution of 1highG is confirmed again by comparing the molecular junction elongation length of 1highG (∼0.25 nm) with that of a previously reported **A1** junction (∼0.17 nm, illustrated in Figure S4),^27^ where the Au electrode is directly attached to an sp^2^ carbon on the benzene ring. We note that the measured junction elongation length for 1highG and 3G (∼0.25 nm and ∼0.29 nm) are in agreement with the calculated junction length (0.82 and 0.89 nm, defined as the distance between the two Au atoms that are in contact to the molecule), after consideration of the ∼0.7 nm snap back distance.^52–54^ Additionally, in comparison to the previously reported conductance value of a molecule consisting of CH_2_–benzene–CH_2_ backbone terminated with thiomethyl linkers,^39^ this observed conductance of 1highG is higher by a factor of 13, indicating that it likely contains a highly conducting C–Au covalent bond. We note that the formation of 1highG from **1** occurs under a low bias voltage of 90 mV and 5 mV as well (Figure S5), highlighting the efficient formation of C–Au covalent bond with this strategy, in comparison to cases where a large applied voltage is needed.^26,40,41^

Next, we investigate 1lowG. We propose that 1lowG is from the junctions of the homocoupled dimer, **2** (chemical structure in Figure 1b and the junction illustration in Figure 2e), a product formed in air from C–C coupling reactions (Figure S1 in the Supporting Information). The dimer product is attached to both gold electrodes through dative bonds between the sulfur atoms of the thiomethyl groups and Au electrodes, in agreement with the conductance measured for ex-situ-synthesized **2**, which we will discuss next.

To understand the impact of the presence of −CN substituents on the junction conductance, we perform first-principles density functional theory (DFT) calculations of 1highG and 3G junctions (details are given in the Methods section). Since we have a covalent C–Au linkage in 1highG and 3G junctions, we first focus on the structural aspects, i.e., the dihedral angle between the C–Au bond and the phenyl plane^34,42^ and the C–Au bond length.^43^ In optimized junction structures (Figure 3a and 3b), we see that both junctions exhibit a close to 90° dihedral angle between the C–Au bond and the phenyl plane, in agreement with a previous study.^34^ **1** and **3** also show similar rotational barriers for the C–Au bond around the phenyl ring (5.77 kcal/mol for **1** and 7.38 kcal/mol for **3**; see Figure S6), suggesting that the presence of the −CN substituents does not alter the molecular conformation at the C–Au interface. We find a larger C–Au bond length for **1** (2.24 Å) in comparison to **3** (2.13 Å). Overall, the calculated transmission near Fermi level is larger for **3** than for **1** (Figure 3c), with **3** displaying a broadened gateway state that is often seen for covalent bonds such as C–Au and S–Ag.^36,44^ DFT calculations indicate that the presence of the −CN groups lower the conductance, in agreement with the experimental results.

**Figure 3:**
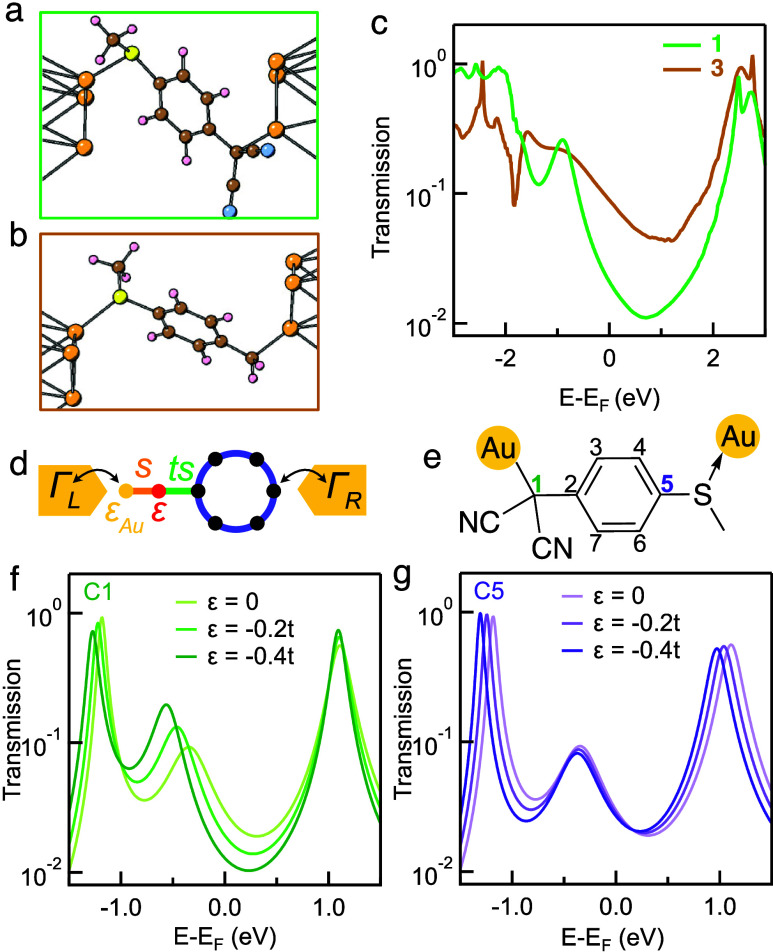
Transmission calculations of 1highG and 3G molecular junctions. The optimized structures of (a) 1highG and (b) 3G junctions using DFT. (c) Transmission functions for 1highG and 3G junctions calculated using DFT. (d) A scheme of the tight-binding modeling of the 1highG junction. (e) A structure of the 1highG junction showing the labeled carbon positions. Transmission functions for the junction shown in panel (d) with a varying on-site energy for (f) C1 and (g) C5 calculated using a tight-binding model with the best-fit parameters.

We do no find significant differences in the junction structures between 1highG and 3G, which motivates us to analyze the electronic effect of the electron-withdrawing −CN groups. To do this, we employ a tight-binding model where the on-site energy ε of the carbon (Figure 3d) represents the electronic effect from the substituents on this carbon.^45^ To model the covalent benzyl-Au linkage, we adopt a coupling s and on-site energy ε_Au_ for the C–Au gateway state, as illustrated in Figure 3d (details are given in Supporting Information Section V).^36^ Among the seven carbon positions as shown in Figure 3e, we find that only when we vary the on-site energy for C1, we see a clear change in the transmission value near *E*_F_ (Figure 3f), largely due to a shift in the position of the gateway state. For C2–C7, in contrast, we do not see any visible changes in the transmissions near *E*_F_ (Figure 3g and Figure S7). This result emphasizes that the substituents on the carbon at the C–Au linkage bond can effectively tune the charge transport by a factor of 10, which is significantly enhanced, in comparison to the effect from substituents on other carbon positions in the backbone.^46^ For example, when we compare the conductance for the dimer **2** with the compound having the four −CN groups replaced by hydrogens, the conductance (4.0 × 10^–5^ G_0_ vs 1.6 × 10^–5^ G_0_)^35^ largely remains the same.

We further synthesize **2** by oxidizing **1** with potassium ferricyanide under aqueous basic conditions. Two conductance peaks, located at ∼8.5 × 10^–3^ G_0_ and ∼7.1 × 10^–5^ G_0_, with corresponding plateau lengths of ∼0.27 nm and ∼ 0.51 nm, are seen in the conductance experiment **2** (Figure 4b and Figure S8a), in agreement with the results of **1**. We thus propose the same junctions for 2highG and 2lowG as those for 1highG and 1lowG, as illustrated in Figure 4a. In contrast to the spontaneous reaction of **1** in solution, we note that, in the high-performance liquid chromatography (HPLC) analysis of **2** after the solution of **2** is left in air for 300 h or on a rough Au surface for 4 h, no peak corresponding to **1** is seen (Figure S9). Since we assume that **2** undergoes homolytic cleavage reaction to form the radical of **1** in STM-BJ experiments, we add 10 equiv of cyclohexane into the solution of **2** (2* in Figure 4c) to suppress this reaction.^47,48^ Specifically, cyclohexanes surrounding **2** form a nonpolar “cage” that facilitates geminate recombination of the radical pair generated by homolytic cleavage of **2**, thereby preventing their dissociation into free radicals. The absence of 2highG in the conductance histogram of 2* (Figure 4d and Figure S8b) confirms our hypothesis.

**Figure 4:**
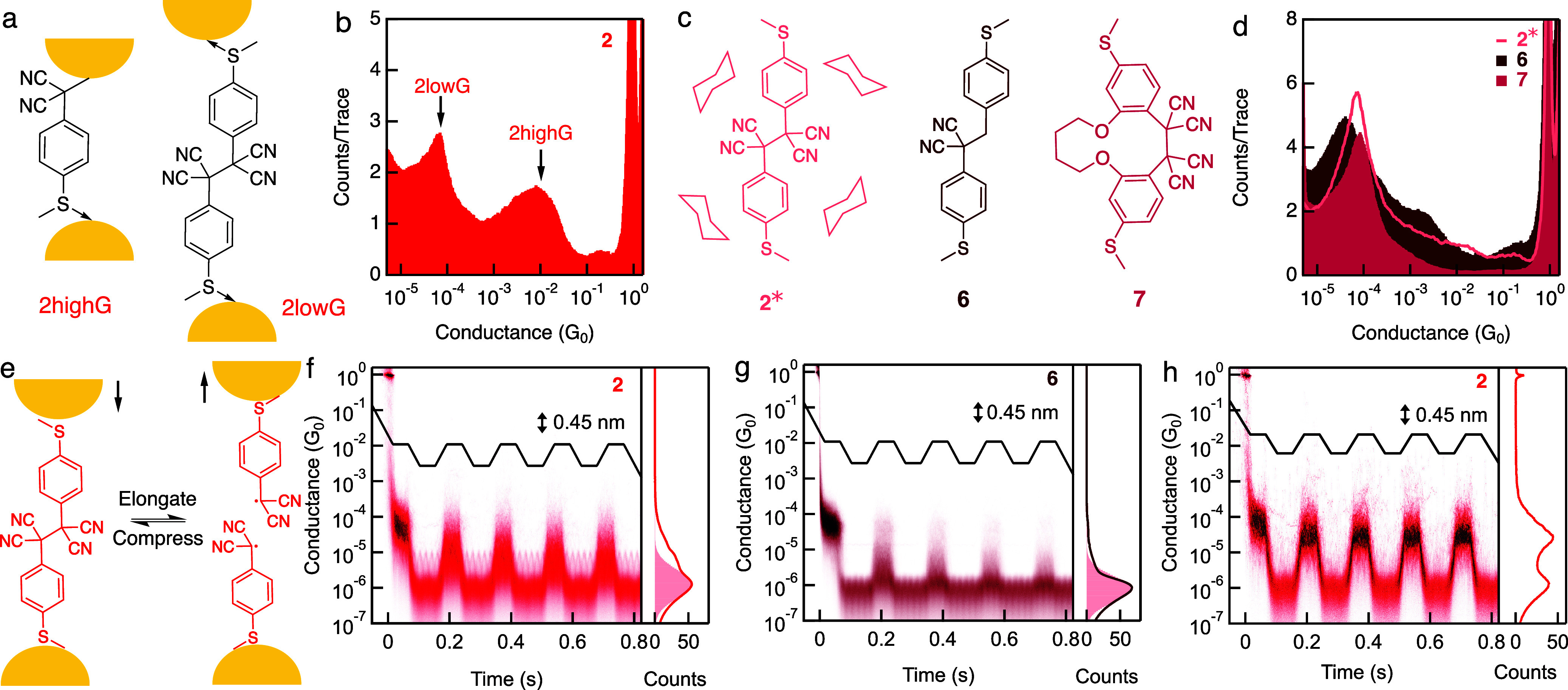
In-situ reaction of **b** in forming **1** radical junctions in break-junction experimentss. (a) Schematic illustration of molecular junctions formed in STM-BJ experiments of **2**. (b) 1D histogram of **2** measured in TCB under 540 mV. (c) Schematic illustration of 2* (solution of **2** with addition of cyclohexane), and chemical structures of **6** and **7**. (d) 1D histograms of 2*, **6**, and **7** measured in TCB under 540 mV. For 2*, 10 equiv of cyclohexane was added in the solution of **2**. (e) Schematic illustration of the mechanically controlled switching of **2**. (f–h) 2D conductance histograms constructed from traces in which (f and g) a molecular junction was held in the first hold period or (h) a molecular junction was held in both the first and the last hold periods. Panels (f) and (h) show the data of **2** and (g) shows the data of **6** for comparison. The black curves show the piezo ramps applied and a distance of 0.45 nm was applied to elongate or compress the junction in each cycle. Right panels in panels (f)–(h) show the corresponding 1D conductance histograms constructed from the data spanning from the end of first hold to the end of final hold, except in panel (h), where the data from the first hold is included.

We further design and synthesize compounds **6** and **7**. Both compounds show only one conductance peak near that of 2lowG (see Figure 4d and Figures S8 and S10), confirming that 2lowG is from junctions of **2** attached to Au through S→Au dative bonds. The central C­(sp^3^)–C­(sp^3^) bond in **6** is not cleaved as benzyl radicals are not as stable as aryl dicyanomethyl radicals. In **7**, the two phenyl rings are additionally connected by a −O–(CH_2_)_4_–O– bridge (Figure 4c), and we find that the cleaved radical junction is also not seen in the measurement of **7**. We suggest that upon C–C bond cleavage in **7**, if it occurs, two aryl dicyanomethyl radicals are linked. The flexible bridge confines the two cleaved fragments in close proximity, thereby enhancing the probability of weak association between the two radicals and reducing the C–Au contact formation.

Given the dynamic nature of the C­(sp^3^)–C­(sp^3^) bond in **2**, which bears a strong electron-donating *para*-thiomethyl group,^49^ we seek to exploit the sub-Ångström control of the single-molecule junction by the STM-BJ technique and mechanically break and reform this bond, as illustrated in Figure 4e. After an initial withdrawal, the tip is then held in place for 50 ms, after which the junction length is elongated and compressed by 0.45 nm for 4 repeating cycles, and each elongation or compression of the junction is followed by a 50 ms hold period. The corresponding piezo displacement trajectories are indicated by the black curves in Figure 4f–h. We select conductance traces that exhibit a molecular junction conductance during the first hold period and use these traces to construct 2D histograms. We find that **2** shows clear conductance distributions above the noise level after a compression from a broken junction (Figure 4f), and in contrast, the control molecule **6** does not (Figure 4g). This difference is clear in the 1D conductance vs. intensity histograms compiled from data spanning from the end of the initial hold period to the end of the final hold period (right panels in Figure 4f and 4g). Additional intensity above the instrument background conductance is seen for **2** but not for **6**.

We next select traces that maintain a molecular junction in both the initial and final hold periods and find that ∼15% and ∼1% of the traces for **2** and **6**, respectively, are selected from the traces that show a molecular junction only in the initial hold (Table S3). This contrasting result reveals that a repeated mechanical cleavage and formation of the C­(sp^3^)–C­(sp^3^) bond is realized in **2**, but not possible in a control structure **6**. Specifically, for **2**, a clear reversible switching between two distinct conductance states with an on/off ratio of ∼150 is seen (Figure 4h), in which the on state corresponds to the single-molecule junction of **2**, and the off state is our instrument background. We find that such regulation between a radical pair and a dimer junction induced by a mechanical modulation is also observed in **7** (Figure S11 and Table S3). We note that we cannot exclusively demonstrate a mechanical modulation between 2highG and 2lowG, as the same conductance switching is also observed in control molecules **6** and 4,4′-ethylenedianiline, when no radical junctions can be formed (Figures S12 and S13).

We further find that when a methylene group is inserted between the phenyl ring and the dicyanomethyl (compound **8**, Figure 5), which is expected to attenuate the electron-donating effect of the phenyl ring toward the radical center and ultimately affect the stability of the reaction intermediates, the homocoupling reaction still proceeds in break junction experiments (Figure 5 and Figure S14). A similar reaction was not achieved through other conventional synthetic attempts that we have made. A detailed discussion is given in Section III in the Supporting Information.

**Figure 5:**
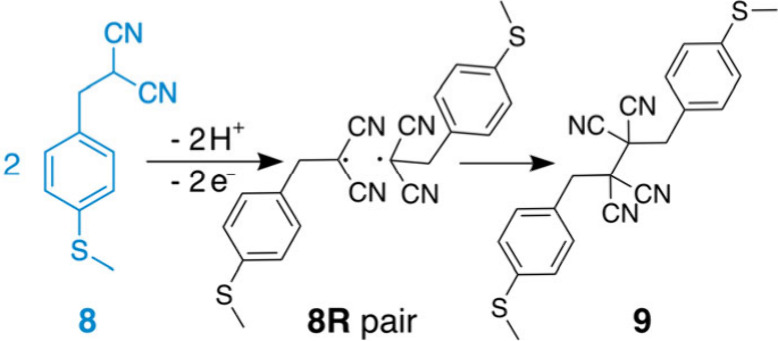
Reaction scheme of **8** in forming **8** radical and dimer **9**. The radical and dimer junctions are observed in the break-junction experiments (Figure S14).

## Conclusions

By designing compounds that generate captodative radicals, we show two conductance states in the break-junction measurements of monomer and in measurements of dimer, highlighting that both monomer and dimer are capable of forming a highly conducting junction of an aryl dicyanomethyl radical linked to Au by C–Au covalent bond, and a dimer junction with low conductance. First-principles calculations further showcase the effectiveness of the –CN substituents in modulating the junction conductance when the substituents are at the linkage carbon position on C–Au. Compression-elongation mechanical modulation experiments demonstrate a reversible conversion between dimers and radical pairs. We further achieve dehydrogenative homocoupling in phenethyl systems by the use of break-junction measurements, when such reactions are not possible through regular synthetic routes. The precise manipulation of chemical bond cleavage and formation at the atomic scale is investigated in this work, enabling new ways to drive and regulate reactions.

## Methods

### Synthesis of **1** and **7′′**

4-Bromothioanisole (2.64 mmol, 1.0 equiv.) or 1,4-bis­(2-bromo-5-(methylthio)­phenoxy)­butane (1.0 mmol, 1.0 equiv) and Pd­(PPh_3_)­Cl_2_ (10 mol%) was added to a prestirred solution of malononitrile (1.9 equiv) and NaOtBu (3.35 equiv) in xylenes (0.2 M) under an inert atmosphere. After 3 h of heating at 130 °C, the reaction was cooled, quenched by HCl (aq. 10%) and purified by flash column chromatography. Compound **1** was obtained in 87% yield and **7′′** in 48% yield.

### Synthesis of **2** and **7**

A prestirred solution of K_3_[Fe­(CN)_6_] (5 equiv) in 10 mL of KOH (aq. 0.3 M) was added to a solution of 1 (0.6 mmol, 1 equiv) in 10 mL of DCM under inert atmosphere. The reaction was stirred for 15–20 min, opened to air. Compound **2** was purified by flash column chromatography in 87% yield and **7** in 36% yield.

### Synthesis of **3**

Details are provided in a previous work.^28^

### Synthesis of **4**, **6**, **8**, and **10**

Substituted malononitriles (1 mmol) were dissolved in acetone (5 mL). Then, an alkyl halide (2 mmol) and K_2_CO_3_ (2.5 mmol) were added. The mixture was stirred overnight at room temperature. After filtration and concentration, the residue was purified by flash chromatography to yield the desired product.

### Conductance Experiments

Single-molecule conductance measurements were conducted using a custom-built scanning tunneling microscope-break junction (STM-BJ) technique that has been described in detail in the Supporting Information of a previous work.^50^ Au substrates were prepared by the use of a thermal evaporation system (Beijing Technol Science Co., Ltd.) with a high-purity Au target (99.999%, Shijiazhuang Huake Metal Materials Technology Co., Ltd.). Au was deposited onto polished steel substrates (⌀ = 15 mm) at a deposition rate of ∼1 Å/s for obtaining ∼100-nm-thick Au films. Prior to each measurement, the prepared Au substrates were treated with UV-ozone (L2002A2-UK, Ossila Limited) for 20 min. A mechanically cut Au wire (⌀ = 0.25 mm, 99.999%, ZhongNuo Advanced Material (Beijing) Technology Co., Ltd.) was used as the STM tip. 0.1–0.5 mM solutions of target molecule in 1,2,4-trichlorobenzene (TCB) were dropped onto the substrate for conductance measurements. The tip was displaced at a speed of ∼19 nm/s and the current and voltage data were acquired at 40 kHz acquisition rate for all measurements. In each measurement, we continuously collected 8000–19 000 conductance traces. **5** (Leyan, 98.11%), 4,4′-ethylenedianiline (Aladdin, ≥97%), cyclohexane (Energy Chemical, 99.5%), and TCB (Aladdin, Spectrophotometric grade, ≥99%) were used without any further purification.

### Mechanical Modulation Experiments

For the conductance traces collected from experiments in which a modified piezo ramp was applied, we determined the average conductance in the initial 50 ms hold period. If this average conductance was within the conductance distribution of this target molecule (2.0 × 10^–5^–2.0 × 10^–4^ G_0_ for **2**, **7**, and 4,4′-ethylenedianiline, and 1.6 × 10^–5^–1.6 × 10^–4^ G_0_ for **6**), we determined that, in this conductance trace, a single-molecule junction of the target molecule was held during the initial hold period. We applied the same method for determining a molecular junction being held during the last hold period. For constructing the 2D histograms, the selected conductance traces were aligned to zero second along the time axis when the first hold period began.

### High-Performance Liquid Chromatography (HPLC) Experiments

HPLC analysis was performed on a 1260 II Agilent system equipped with an UV detector of 210 nm wavelength. The mobile phase had a hexane:isopropanol ratio of 90:10 (v/v). The injection volume was 20 μL. The sample was prepared by dissolving the target molecule in hexane or isopropanol at a concentration of 0.05 mg/mL, and the sample was filtered before injection.

### DFT-Based Transmission Calculations

The geometric optimization of molecules **1** and **3** was first performed using density functional theory (DFT) with the Perdew–Burke–Ernzerhof (PBE) exchange–correlation functional in the generalized gradient approximation (GGA), as implemented in the Quantum ATK X-2025.06 software. In the geometric optimization of **3**, the *n*-butyl-substituted organotin (−Sn­(*n*-Bu)_3_) group was replaced by a hydrogen atom (H) to expedite calculations. Next, single-molecule junction devices were built by bridging the optimized molecules between two Au(111) electrodes. Each electrode was modeled with a six-layer Au(111) slab, where the surface region was shaped into trimers as the binding motif.^51^ Each layer consisted of 4 × 4 Au atoms. The left electrode was coordinated by the terminal S atom of the molecule, while the right electrode was covalently bonded with C atom at the benzyl position (Figure 3a and 3b). During geometry relaxation of the device, the outermost three atomic layers of the left electrode were fixed, and the outermost three layers of the right electrode were treated as a rigid body. All inner atoms in the molecule sandwiched between the two electrodes were allowed to fully relax until all forces were below 0.04 eV/Å. The geometry optimization and transmission calculation of the devices were implemented in Quantum ATK X-2025.06 with the GGA-PBE functional and Fritz-Haber-Institute (FHI) pseudopotentials. A single-ζ polarized (SZP) basis set was applied to Au atoms, and a double-ζ polarized (DZP) basis set was used for all other atoms.

### DFT-Based Coordinate Scan of the Dihedral between C–Au Bond and the Phenyl Plane

One Au atom was used on the side of the C–Au linkage as a proxy for the gold electrode and for determining the Au–C1–C2–C3 dihedral angle; no Au atoms were present on the thiomethyl linker side. The dihedral angle between the C–Au bond and the phenyl plane was constrained while the rest atoms were fully relaxed before the total energy was calculated for each fixed dihedral angle conformation. The calculations were performed using DFT within the Quantum ATK X-2025.06 software package.

### Transmission Calculations with Tight-Binding Models

The tight-binding model provides a simple theoretical framework for describing the charge transport through molecular junctions formed by **1** and **3**. In this model, the π-conjugated backbone is represented by discrete sites with on-site energies and nearest-neighbor hopping integrals. The coupling coefficients between the molecule and the electrodes are modeled by Γ_L_ and Γ_R_, and the C–Au covalent linkage is further described by the covalent gateway site of coupling parameter *s* and on-site energy ε_Au_. The influence of the –CN substitution groups in **1** is modeled as a perturbation that shifts the on-site energy ε of the backbone carbon directly bonded to −CN groups. The transmission function T­(E) is computed using the nonequilibrium Green’s function (NEGF) formalism.

## Supplementary Material



## Notes

Synthetic procedures and characterization of compounds, additional figures and tables, discussion of compound **8**, NMR data, and details of the tight-binding model (PDF)
