# Supplementary material for: Captodative Radicals Enable the Coexistence of Monomer and Dimer Single-Molecule Junctions with 100-Fold Difference in Conductance
Source: J Am Chem Soc. 2026 Jun 9;148(24):24768–75. doi: 10.1021/jacs.6c01727 (PMC13307369; doi:10.1021/jacs.6c01727)
Supplement: Supplementary file 1 [file ja6c01727_si_001.pdf]

Supporting Information

Captodative Radicals Enable the Coexistence of Monomer and Dimer Single-Molecule Junctions with 100-Fold Difference in Conductance

Weiyi Guo,[a,†] Shuai Yao,[b,†] Xueling Xu,[a] Taotao Lu,[c] Chaochao Xie,[c] Yaxuan Zhang,[d] Jiahao Wang,[b] Hongliang Chen,[d] Xuefeng Tan,[c] Yangyang Shen,[b,*] Haixing Li[a,*]

[a]Department of Physics, City University of Hong Kong, Kowloon 999077 Hong Kong SAR, China.

[b]Frontier Institute of Science and Technology, Xi'an Jiaotong University, Yanxiang Road 99, Xi'an 710045, China.

[c]Department of Chemistry, City University of Hong Kong, Kowloon 999077 Hong Kong SAR, China.

[d]Department of Chemistry, Stoddart Institute of Molecular Science, and ZJU-Hangzhou Global Scientific and Technological Innovation Center, Zhejiang University, Hangzhou 310027, China.

[†]Contributed equally

*Corresponding authors: ys3215@xjtu.edu.cn, haixinli@cityu.edu.hk

Table of content

I. Synthetic procedures and characterization of compounds S1
II. Additional figures and tables... S5
III. Discussion of compound **8**.. S11
IV. NMR data S12
V. Details of the tight-binding model S21
VI. References S23

I. Synthetic procedures and characterization of compounds

Synthetic Procedures

General Procedure for linking bromophenol[1]:

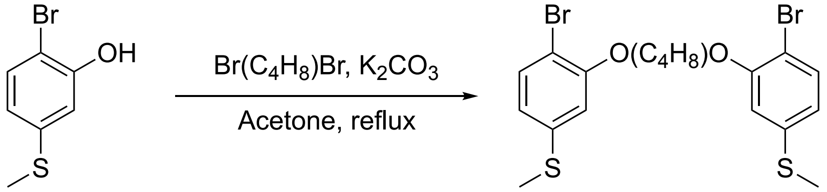

To a 50 mL round-bottom flask, equipped with a stir bar, was added 2-bromo-5-(methylthio)phenol (1.1 g, 5 mmol), dried acetone (15 mL), 1,4-dibromobutane (0.54 g, 2.5mmol), and K_2CO_3 (2.070 g, 15 mmol). The reaction mixture was refluxed for 24 h. The mixture was filtered and washed with acetone. The solvent was removed by rotary evaporation. The crude product was purified by recrystallization to afford **7'**. **1,4-Bis(2-bromo-5-(methylthio)phenoxy)butane(7'):** The general procedure produced a blue solid (0.886g, 72%), ^{1}H NMR (400 MHz, $CDCl_3$) δ 7.42 (d, *J* = 7.3 Hz, 2H), 6.80 (d, *J* = 1.8 Hz, 2H), 6.70 (d, *J* = 8.3 Hz, 2H), 4.14 – 4.12 (m, 4H), 2.47 (s, 6H), 2.11 – 2.08 (m, 4H). ^{13}C NMR (101 MHz, $CDCl_3$) δ 155.3, 139.1, 133.3, 119.6, 111.7, 108.7, 68.7, 25.9, 16.1. HRMS (ESI) m/z calcd. for $C_{18}H_{21}Br_2O_2S_2$ $[M+H]^+$: 492.9324, found: 492.9322.

General procedure for Coupling of Malononitrile to Aryl Bromide[1]:

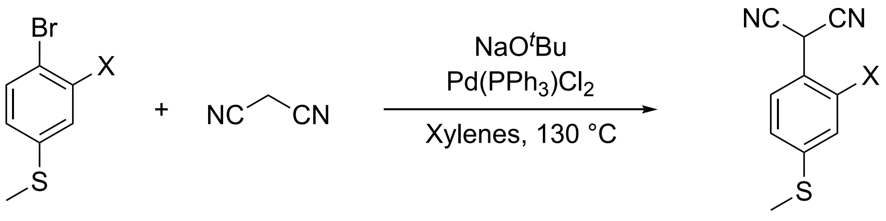

To a dried 50 mL three-neck round bottom flask, which was equipped with a magnetic stirring bar, was added malononitrile (0.21 mL, 3.8 mmol), NaO*t*Bu (0.644 g, 6.7 mmol) and xylenes (10 mL) and stirred under a nitrogen atmosphere for 0.5 h at room temperature. Aryl bromide (2mmol) and $Pd(PPh_3)_2Cl_2$ (0.100 g) were added and the reaction was stirred at 130 °C for 3 h. The reaction mixture was cooled down to room temperature and quenched by 10% HCl aqueous solution. The resulting mixture was filtered through celite and extracted with CH_2Cl_2 (3 × 50 mL). The organic layers were combined and dried with Na_2SO_4, and the solvent was removed by rotary evaporation. The crude product was purified by chromatography on silica gel (hexane: CH_2Cl_2 = 1:1 as eluent).

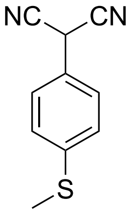

2-(4-(Methylthio)phenyl)malononitrile (1): The general procedure produced a yellow solid (0.422 g, 85%), ^{1}H NMR (400 MHz, $CDCl_3$) δ 7.40 (d, *J* = 8.6 Hz, 2H), 7.33 (d, *J* = 8.6 Hz, 2H), 5.02 (s, 1H), 2.51 (s, 3H). ^{13}C NMR (101 MHz, $CDCl_3$) ^{13}C NMR (101 MHz, $CDCl_3$) δ 142.5, 127.5, 127.1, 122.2, 111.7, 27.7, 15.2. HRMS (ESI) m/z calcd. for $C_{10}H_7N_2S$ $[M-H]^-$: 187.0335, found: 187.0340.

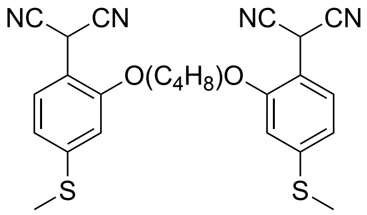

2,2'-((Butane-1,4-diylbis(oxy))bis(4-(methylthio)-2,1-phenylene))dimalononitrile(7”): The general procedure (**7’** was used as aryl bromide) produced a blue solid (0.223g, 48%), ^{1}H NMR (400 MHz, $CDCl_3$) δ 7.38 (d, *J* = 8.1 Hz, 2H), 6.89 (d, *J* = 8.5 Hz, 2H), 6.84 (s, 2H), 5.14 (s, 2H), 4.20 (s, 4H), 2.51 (d, *J* = 0.9 Hz, 6H), 2.16 (s, 4H). ^{13}C NMR (101 MHz, $CDCl_3$) δ 155.5, 144.3, 128.8, 118.2, 111.8, 111.1, 109.7, 68.1, 25.8, 23.6, 15.4. HRMS (ESI) m/z calcd. for $C_{24}H_{21}N_4O_2S_2$ $[M-H]^-$: 461.1111, found: 461.1110.

General Oxidation Procedure[1]:

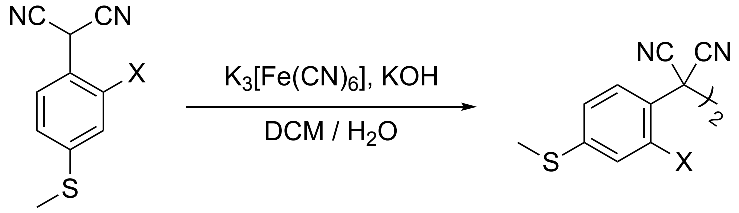

To a 50 mL round bottom flask was added 0.3 mmol of tethered substrate dissolved in 10 mL of dichloromethane. The flask was then sealed and purged on a Schlenk line to introduce nitrogen as an inert atmosphere. In a separate 25 mL round bottom flask was added 3 mmol of $K_3[Fe(CN)_6]$. The flask was then purged and 10 mL of 0.3 M KOH (aq.) was added. This mixture was allowed to mix for 5 minutes before transferal to the 50 mL round bottom. After mixing the oxidant with the substrate the reaction was allowed to proceed for 10-15 minutes. The mixture was exposed to air, extracted with dichloromethane (3 × 20 mL), dried with Na_2SO_4, and the solvent removed by rotary evaporation. Dimmer product was purified by flash column chromatography.

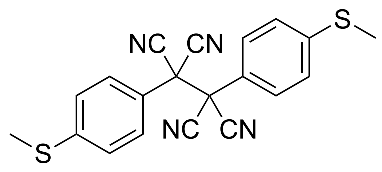

1,2-Bis(4-(methylthio)phenyl)ethane-1,1,2,2-tetracarbonitrile (2): The general procedure produced a white solid (0.104 g, 87%), ^{1}H NMR (400 MHz, $CDCl_3$) δ 7.43 (d, *J* = 8.3 Hz, 4H), 7.31 (d, *J* = 8.4 Hz, 4H), 2.54 (s, 6H). ^{13}C NMR (101 MHz, $CDCl_3$) δ 145.8, 128.5, 125.9, 120.1, 110.3, 29.7, 14.8.

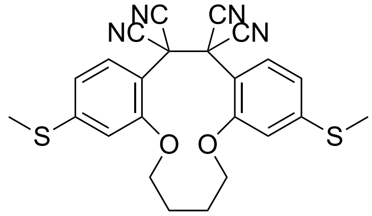

3,12-Bis(methylthio)-6,7,8,9-tetrahydrodibenzo[g,k][1,6]dioxacyclododecine-15,15,16,16-tetracarbonitrile(7): The general procedure produced a blue solid (0.050 g, 36%),^{1}H NMR (400 MHz, $CDCl_3$) δ 7.68 (d, *J* = 8.3 Hz, 2H), 6.97 (d, *J* = 8.3, 1.8 Hz, 2H), 6.87 (s, 2H), 4.35 (s, 2H), 4.12 (s, 2H), 2.55 (s, 6H), 2.30 (s, 2H), 2.20 (s, 2H). ^{13}C NMR (101 MHz, $CDCl_3$) δ 156.1, 147.1, 128.7, 117.5, 111.8, 111.0, 109.8, 109.1, 77.3, 77.0, 76.7, 69.4, 26.8, 15.0. HRMS (ESI) m/z calcd. for $C_{24}H_{21}N_4O_2S_2$ $[M+H]^+$: 461.1101, found: 461.1106.

General procedure for S_N2[2]:

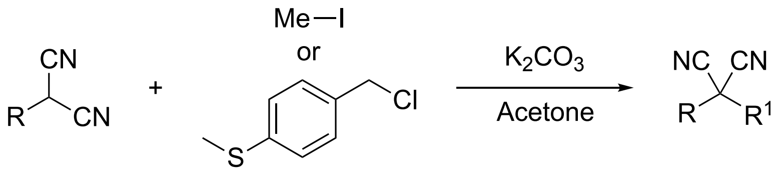

Substituted malononitrile (1 mmol) was dissolved in acetone (5 mL). Then, alkyl halide (2 mmol) and K_2CO_3 (2.5 mmol) were added. The mixture was stirred overnight at room temperature. After filtration and concentration, the residue was purified by flash chromatography to yield desired product.

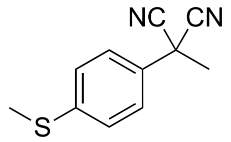

2-Methyl-2-(4-(methylthio)phenyl)malononitrile (4): The general procedure produced a colorless oil (0.186 g, 92%), ^{1}H NMR (400 MHz, $CDCl_3$) δ 7.48 (d, *J* = 8.6 Hz, 2H), 7.32 (d, *J* = 8.6 Hz, 2H), 2.50 (s, 3H), 2.09 (s, 3H). ^{13}C NMR (101 MHz, $CDCl_3$) δ 141.9, 129.3, 126.9, 125.7, 115.6, 36.0, 29.2, 15.2. HRMS

(ESI) m/z calcd. for $C_{11}H_{11}N_2S$ $[M+H]^+$: 203.0638, found: 203.0637.

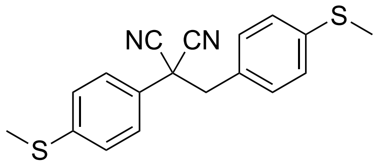

2-(4-(Methylthio)benzyl)-2-(4-(methylthio)phenyl)malononitrile(6): The general procedure produced a white solid (0.034 g, 81%), ^1H NMR (400 MHz, $CDCl_3$) δ 7.34 (d, J = 8.7 Hz, 2H), 7.28 (d, J = 8.6 Hz, 2H), 7.18 (d, J = 8.4 Hz, 2H), 7.04 (d, J = 8.3 Hz, 2H), 3.39 (s, 2H), 2.51 (s, 3H), 2.48 (s, 3H). ^{13}C NMR (101 MHz, $CDCl_3$) δ 141.9, 139.8, 130.8, 127.8, 127.5, 126.6, 126.5, 126.2, 114.6, 48.0, 43.6, 15.4, 15.2. HRMS (ESI) m/z calcd. for $C_{18}H_{17}N_2S_2$ $[M+H]^+$: 325.0828, found: 325.0825.

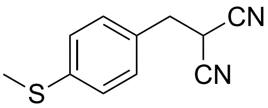

2-(4-(Methylthio)benzyl)malononitrile(8): The general procedure produced a white solid (0.306g, 71%), ^1H NMR (400 MHz, $CDCl_3$) δ 7.28 – 7.22 (m, 4H), 3.88 (t, J = 6.8 Hz, 1H), 3.23 (d, J = 6.8 Hz, 2H), 2.48 (s, 3H). ^{13}C NMR (101 MHz, $CDCl_3$) δ 139.8, 129.6, 129.4, 126.9, 112.2, 36.2, 25.0, 15.5. HRMS (ESI) m/z calcd. for $C_{11}H_9N_2S$ $[M-H]^-$: 201.0491, found: 201.0492.

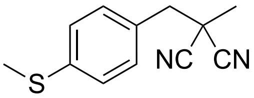

2-Methyl-2-(4-(methylthio)benzyl)malononitrile(10): The general procedure produced a white solid (0.084g, 80%), ^1H NMR (400 MHz, $CDCl_3$) δ 7.28 – 7.24 (m, 4H), 3.14 (s, 2H), 2.47 (s, 3H), 1.77 (s, 3H). ^{13}C NMR (101 MHz, $CDCl_3$) δ 139.8, 130.6, 128.5, 126.5, 116.0, 43.9, 33.3, 24.3, 15.4. HRMS (ESI) m/z calcd. for $C_{12}H_{13}N_2S$ $[M+H]^+$: 217.0794, found: 217.0796.

II. Additional figures and tables

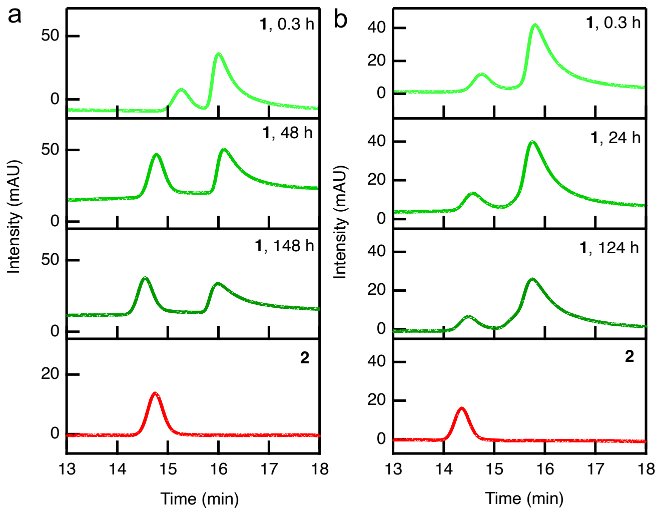

Figure S1. The high-performance liquid chromatography (HPLC) results of 0.05 mg/ml **1** (green) in (a) hexane and (b) isopropanol measured after the solution of **1** has been left in air for 0.3, 48 (24), or 148 (124) hours. The solution was prepared immediately after **1** was taken out of an inert atmosphere glove box. The HPLC results of dilute **2** (red) in (a) hexane and (b) isopropanol are shown for comparison (the solubility of **2** in solvents hexane as well as isopropanol is lower than 0.05 mg/ml).

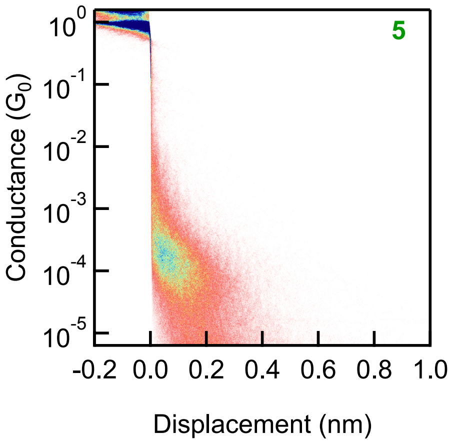

Figure S2. 2D histogram of **5** created from the same data that were used to generate the 1D histogram in Figure 2l in the manuscript.

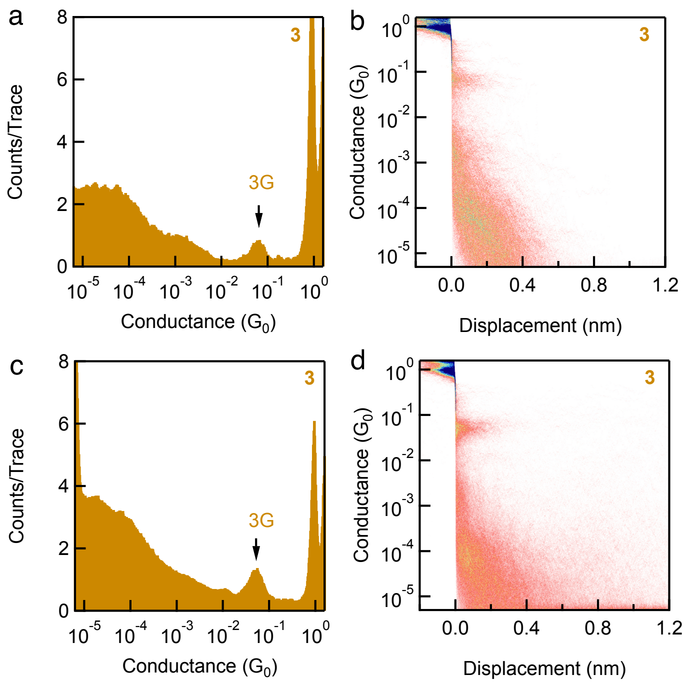

Figure S3. Data of two repeated experiments of **3** measured in TCB under 540 mV.

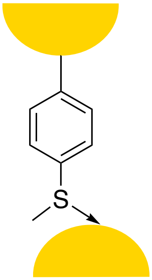

Figure S4. Schematic illustration of the **A1** molecular junction.

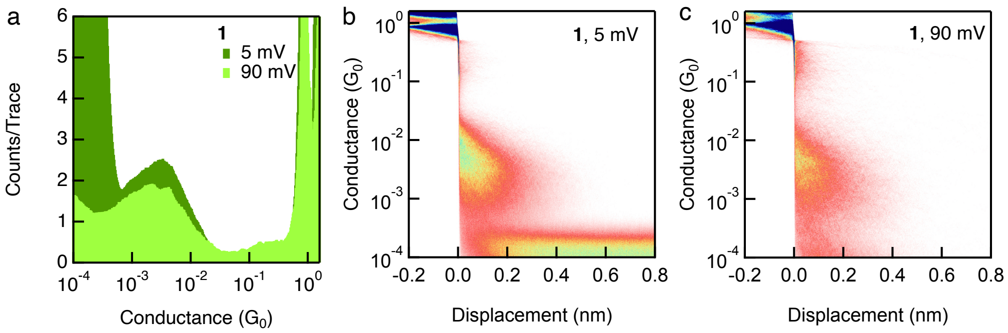

Figure S5. (a) 1D conductance histograms of **1** measured in TCB under 5 mV and 90 mV respectively. 2D conductance histograms of **1** under (b) 5 mV and (c) 90 mV created from the same data that were used to generate the 1D histograms in (a).

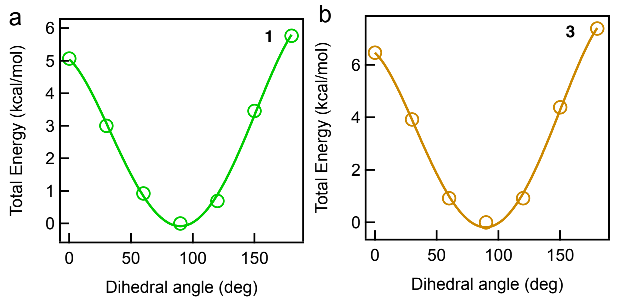

Figure S6. Calculated total energy as a function of the dihedral angle between the C-Au bond and the phenyl plane for (a) **1** and (b) **3** covalently bonded to one Au atom.

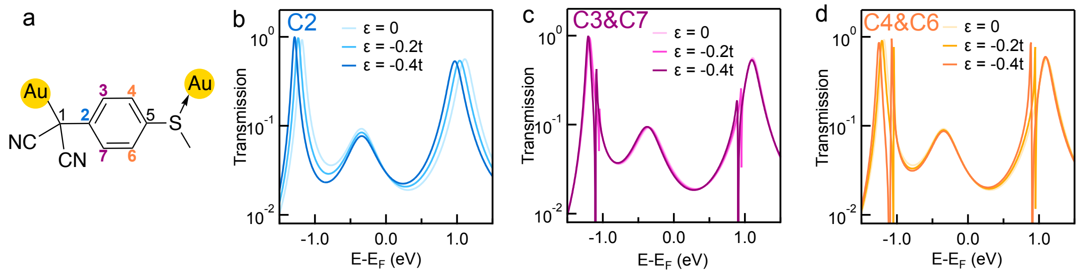

Figure S7. (a) A structure of the 1highG junction showing the labeled carbon positions. Transmission functions for the junction shown in (a) with a varying on-site energy for (b) C2, (c) C3 and C7, and (d) C4 and C6, calculated using a tight-binding model with the best-fit parameters.

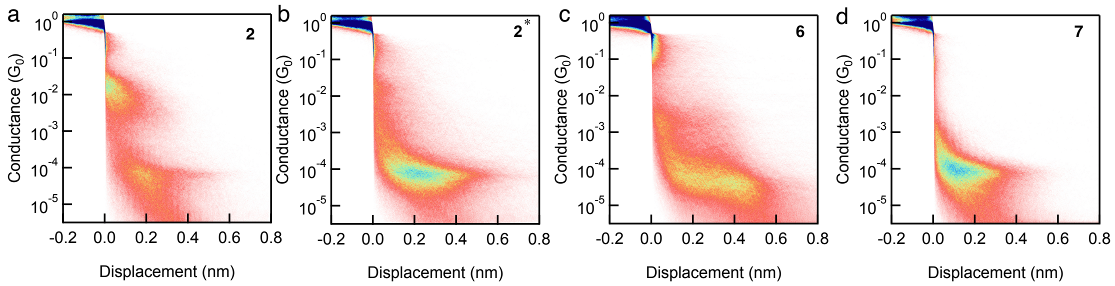

Figure S8. 2D conductance histograms of (a) **2**, (b) **2***, (c) **6**, and (d) **7** under 540 mV created from the same data that were used to generate the 1D histograms in Figure 4 in the manuscript.

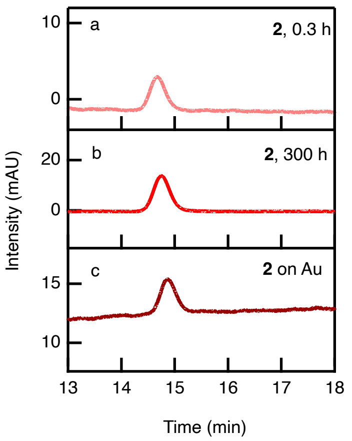

Figure S9. HPLC results of **2** in hexane measured after the solution (a) was left in air for 0.3 hour, (b) was left in air for 300 hours, and (c) was dropped on a Au substrate and left in air for 4 hours.

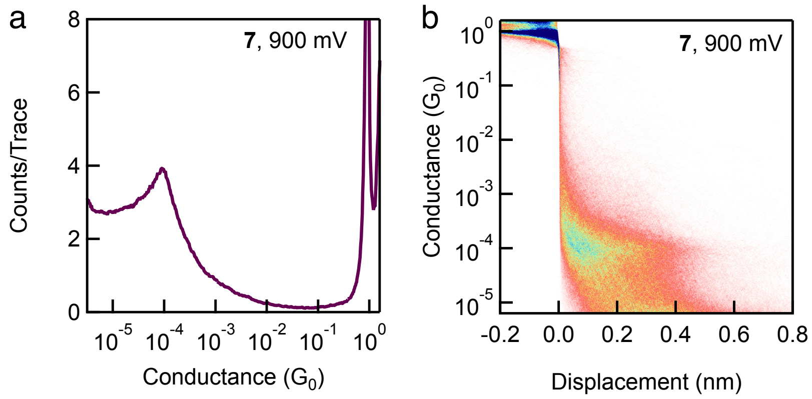

Figure S10. (a) 1D and (b) 2D conductance histograms of **7** measured in TCB under 900 mV.

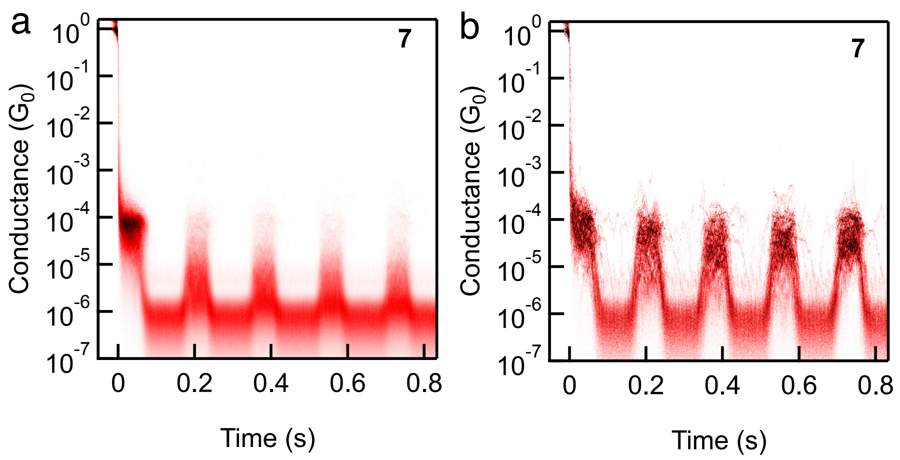

Figure S11. 2D conductance histograms of **7** constructed from traces in which (a) a molecular junction was held in the first hold period or (b) a molecular junction was held in both the first and last hold periods.

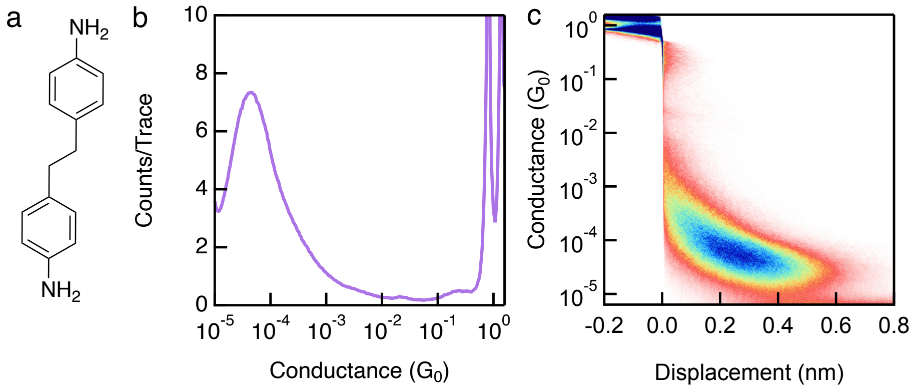

Figure S12. (a) Chemical structure of 4,4'-ethylenedianiline. (b) 1D and (c) 2D conductance histograms of 4,4'-ethylenedianiline measured in TCB under 225 mV.

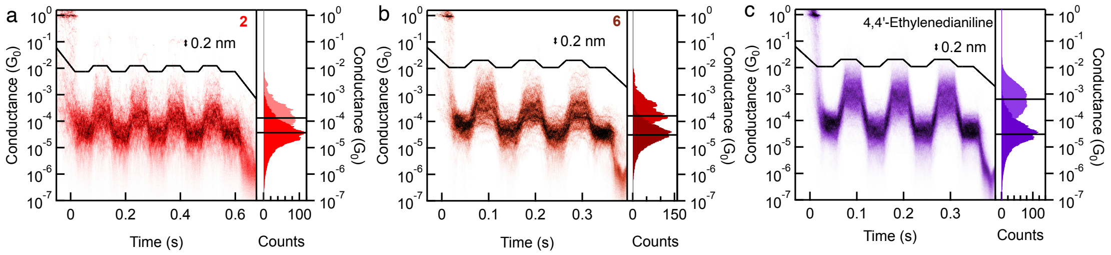

Figure S13. 2D conductance histograms of (a) **2**, (b) **6**, and (c) 4,4'-ethylenedianiline constructed from traces in which a molecular junction was held in both the first and last hold periods. The black curves show the piezo ramps applied and a distance of 0.2 nm was applied to elongate or compress the junction in each cycle. Right panels show the corresponding 1D conductance histograms constructed from the data spanning the penultimate and antepenultimate hold periods, respectively. The solid lines denote the centers of the conductance states.

Reaction time	Area of the peak of reactant **1** (mAU·s)	Area of the peak of product **2** (mAU·s)	Percentage of product **2**
0.3 h	1565.8	390.7	20.0%
48 h	1113.4	648.2	36.8%
148 h	748.7	555.1	42.6%

Table S1. Analysis of the HPLC data presented in Figure S1(a).

Reaction time	Area of the peak of reactant **1** (mAU·s)	Area of the peak of product **2** (mAU·s)	Percentage of product **2**
0.3 h	1421.9	254.6	15.2%
24 h	1271.1	206.1	14.0%
124 h	1067.0	164.5	13.4%

Table S2. Analysis of the HPLC data presented in Figure S1(b).

Compound	# of traces that show a molecular junction conductance in the first hold period	# of traces that show a molecular junction conductance in both the first and last hold periods	Percentage survived*
**2**	2129	308	14.5%
**6**	2921	28	1.0%
**7**	2662	112	4.2%

Table S3. Summary of the number of traces selected for compounds **2**, **6**, and **7** in the mechanical modulation experiments shown in Figure 4 and Figure S11. *Percentage survived is calculated by dividing the value in the third column by that in the second column of this table.

III. Discussion of compound **8**

To investigate whether this reversible dimerization-dissociation reaction is specific to the aryl dicyanomethyl structure, we designed and synthesized compound **8** (chemical structure in Figure 5 in the manuscript). In detail, we perform single-molecule conductance measurement of 0.5 mM **8** in TCB under a 540 mV tip bias. We observe two conductance peaks, labeled as 8highG and 8lowG, at conductance of ~1.6×10^{-3} G_0 and ~2.5×10^{-5} G_0 (Figure S14a), lower than the conductance values for 1highG and 1lowG, respectively. The corresponding molecular junction elongation lengths for 8highG and 8lowG are ~0.31 nm and ~0.78 nm (Figure S14b), larger than those seen for 1highG and 1lowG, respectively. The lack of clear conductance feature for **10** (Figure S14a and S14e) demonstrates again that uncleaved **8** likely cannot bind to Au electrodes in forming molecular junctions. We therefore assign 8highG to junctions formed by radical **8R** (Figure S14c) and 8lowG to junctions formed from homocoupled dimers **9** (Figure S14d). Taken together, the dehydrogenation reaction generating radical **8R** and the subsequent homocoupling of **8R** to form **9** are uniquely catalyzed by STM-BJ experiments. We emphasize that we have not achieved the synthesis of **9** by other synthetic approaches.

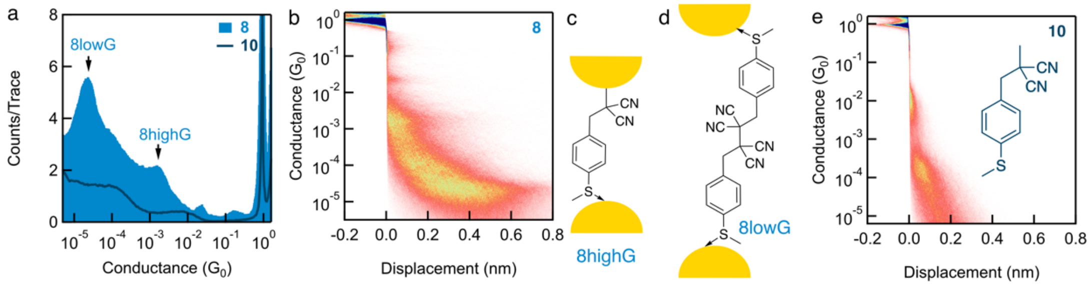

Figure S14. (a) 1D histograms of **8** and **10** measured in TCB under 540 mV. (b) 2D histogram of **8** created from the same data that were used to generate the 1D histogram in (a). Schematic illustration of molecular junctions assigned for (c) 8highG and (d) 8lowG in STM-BJ experiments of **8**. (e) 2D histogram of **10** created from the same data that were used to generate the 1D histogram in (a). Inset: chemical structure of **10**.

IV. NMR data

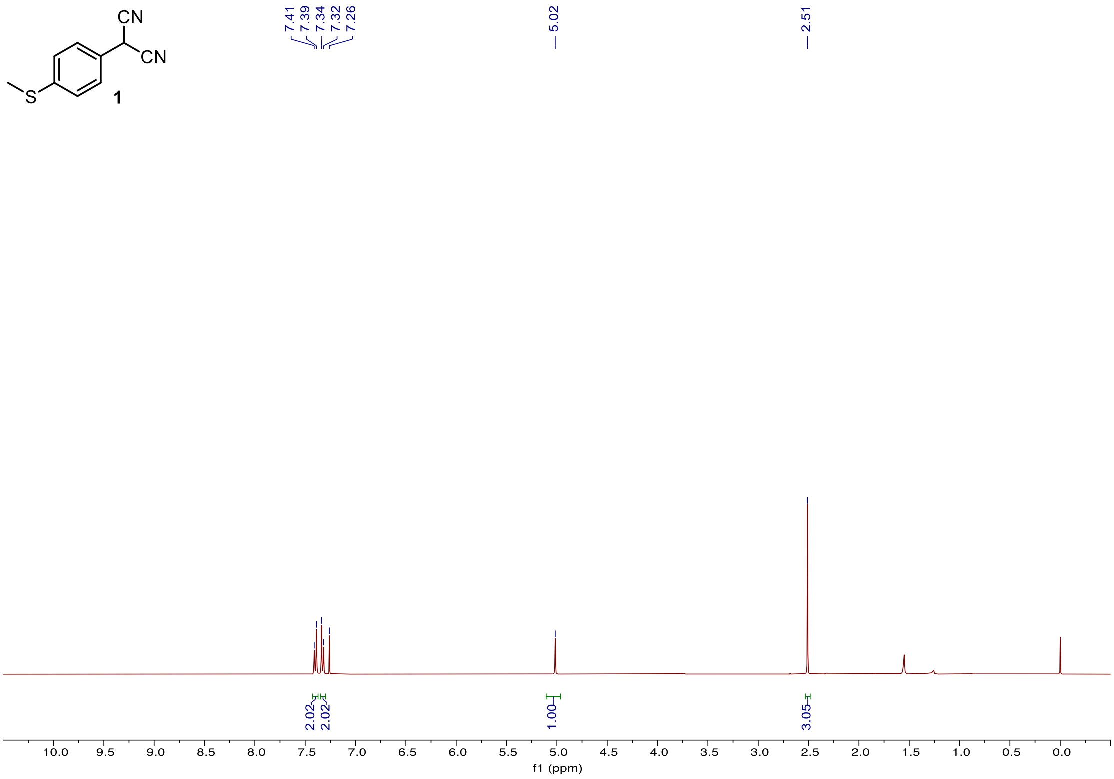

Figure S15. ^{1}H NMR spectrum of compound **1** in $CDCl_3$.

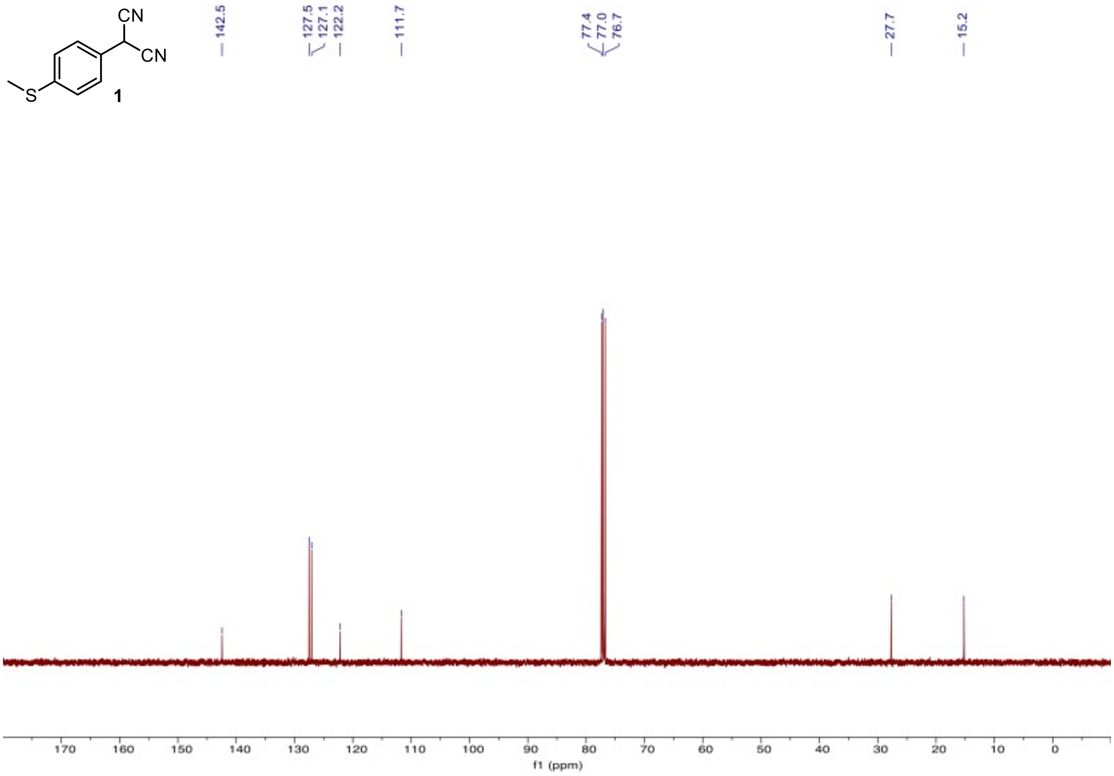

Figure S16. ^{13}C NMR spectrum of compound **1** in $CDCl_3$.

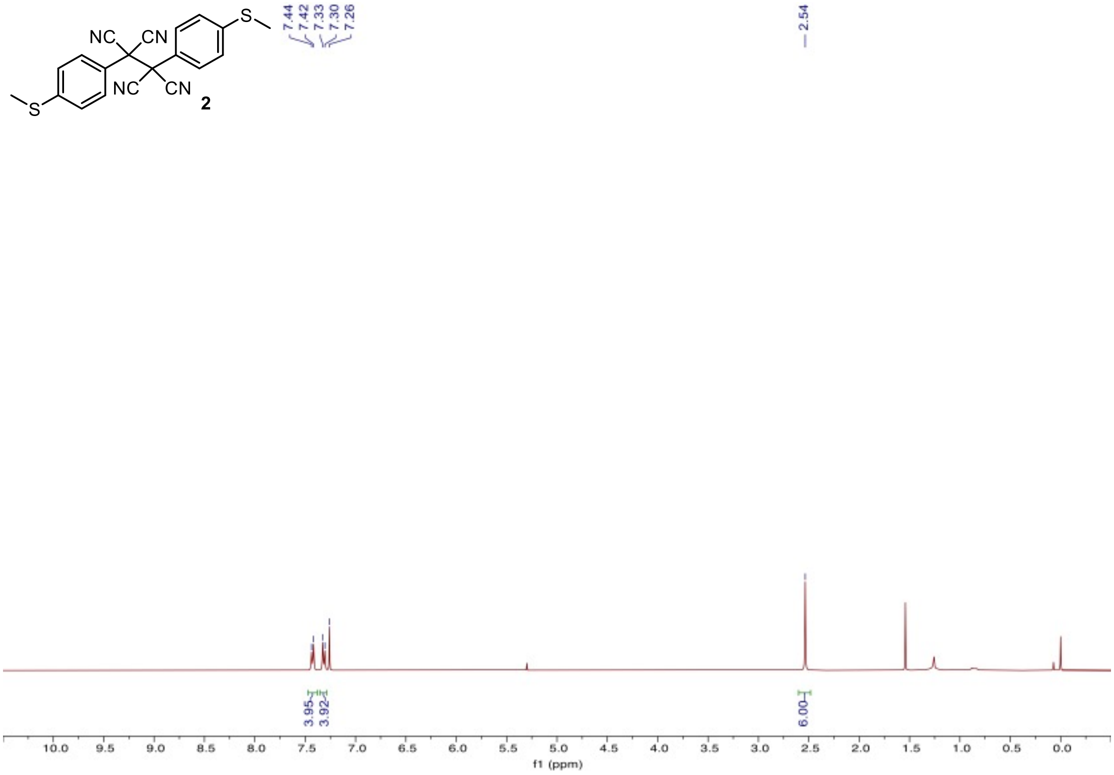

Figure S17. ^{1}H NMR spectrum of compound **2** in $CDCl_3$.

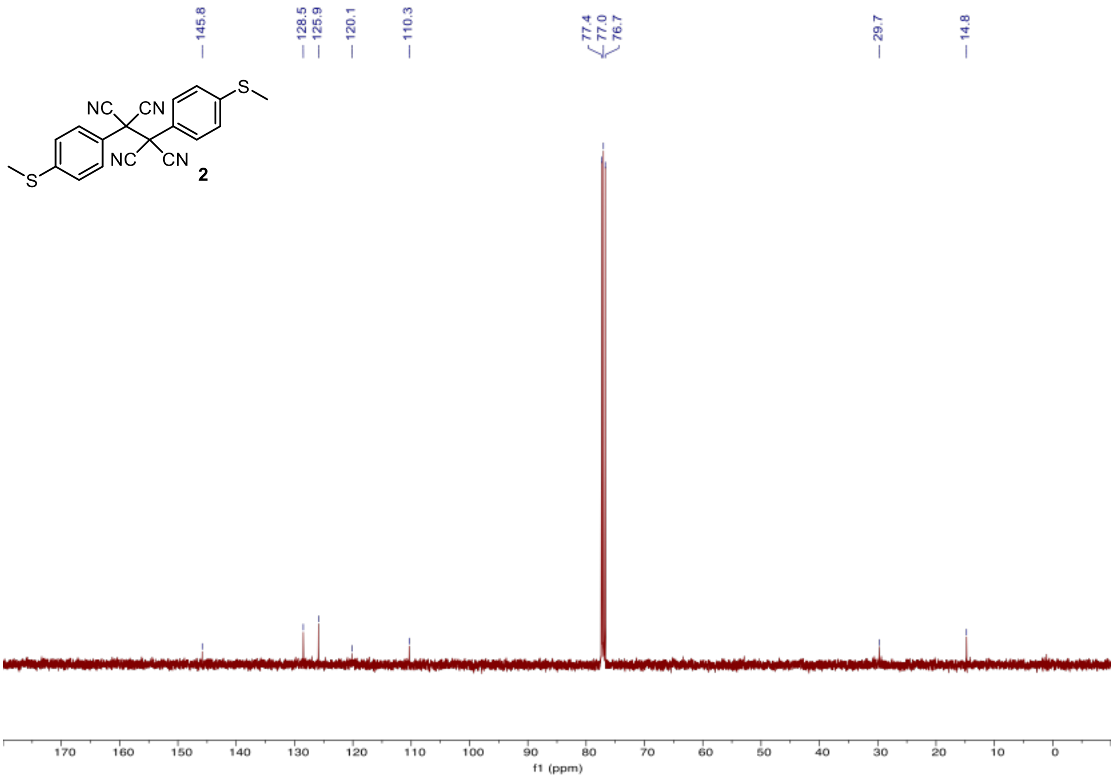

Figure S18. ^{13}C NMR spectrum of compound **2** in $CDCl_3$.

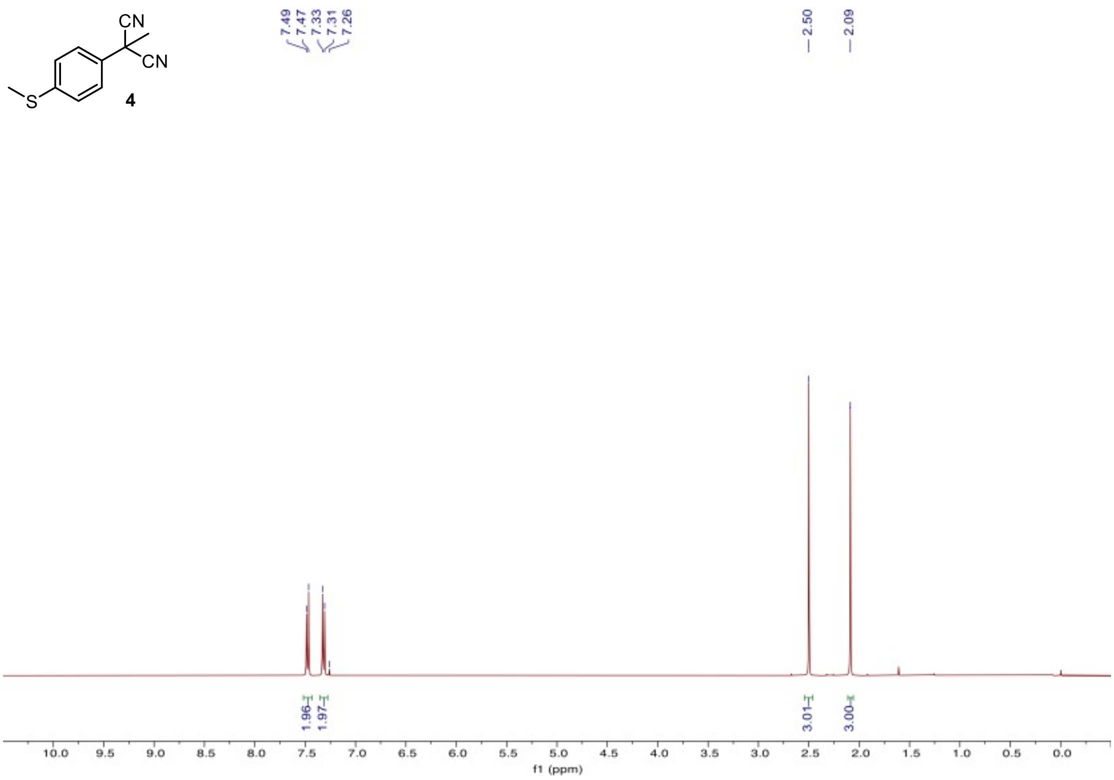

Figure S19. ^{1}H NMR spectrum of compound **4** in $CDCl_3$.

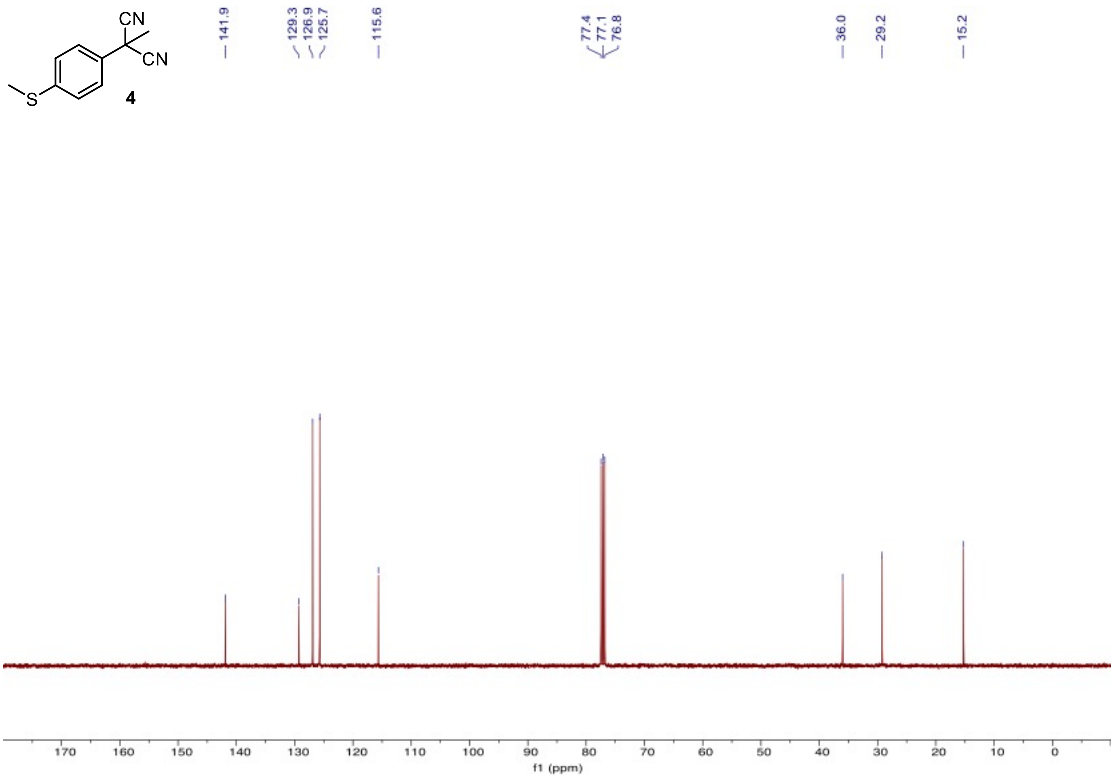

Figure S20. ^{13}C NMR spectrum of compound **4** in $CDCl_3$.

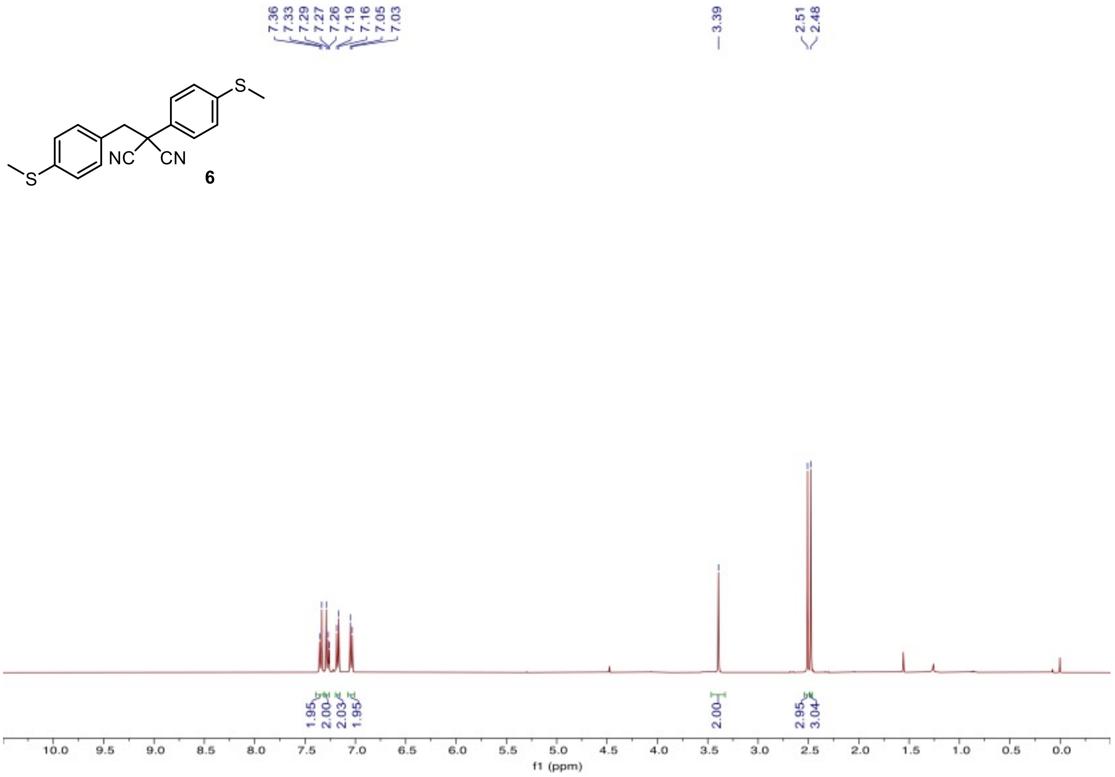

Figure S21. ^{1}H NMR spectrum of compound **6** in $CDCl_3$.

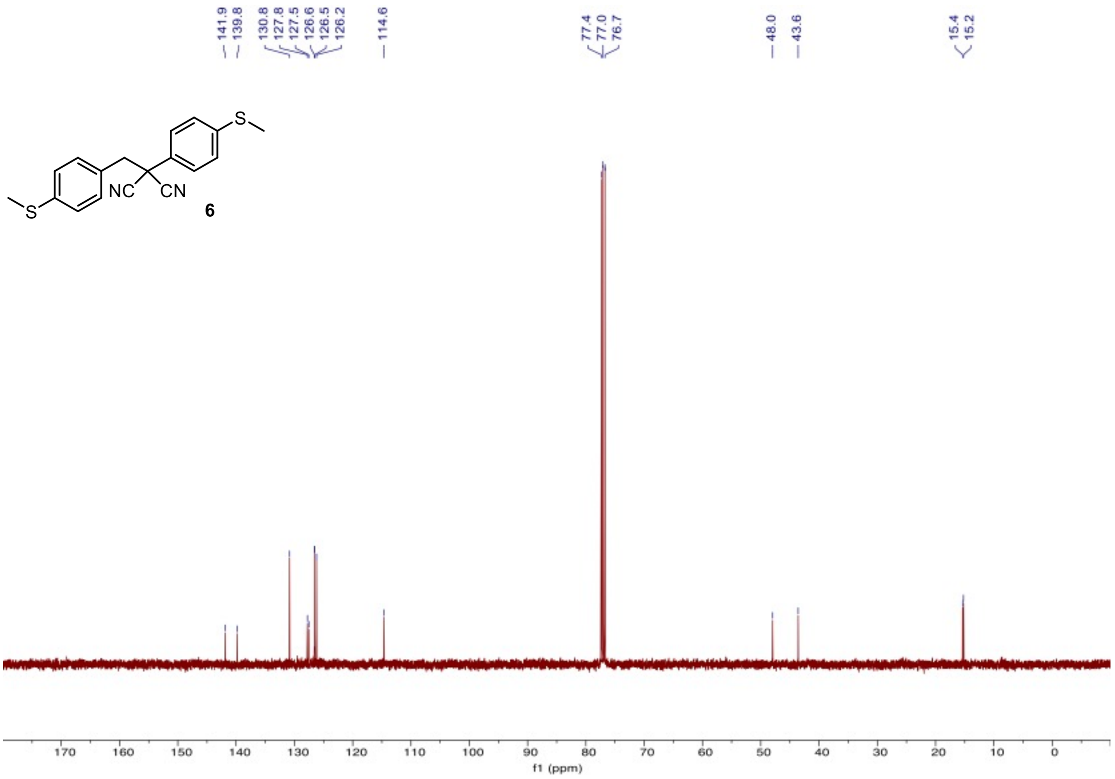

Figure S22. ^{13}C NMR spectrum of compound **6** in $CDCl_3$.

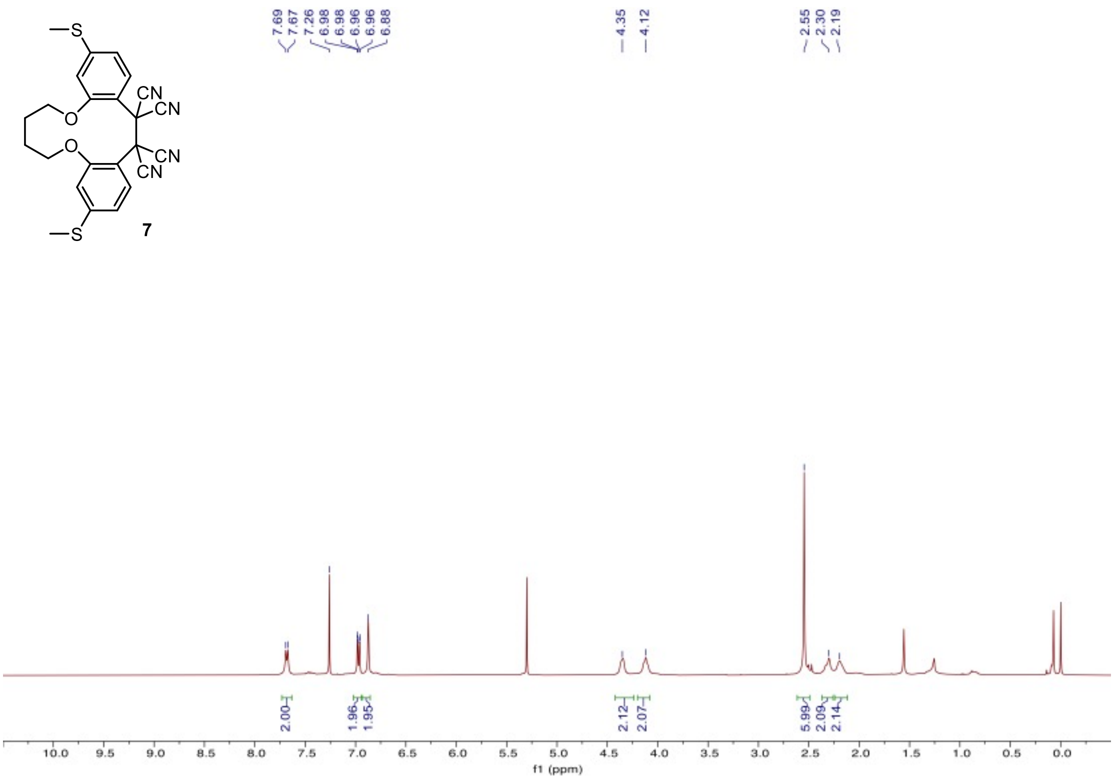

Figure S23. ^{1}H NMR spectrum of compound **7** in $CDCl_3$.

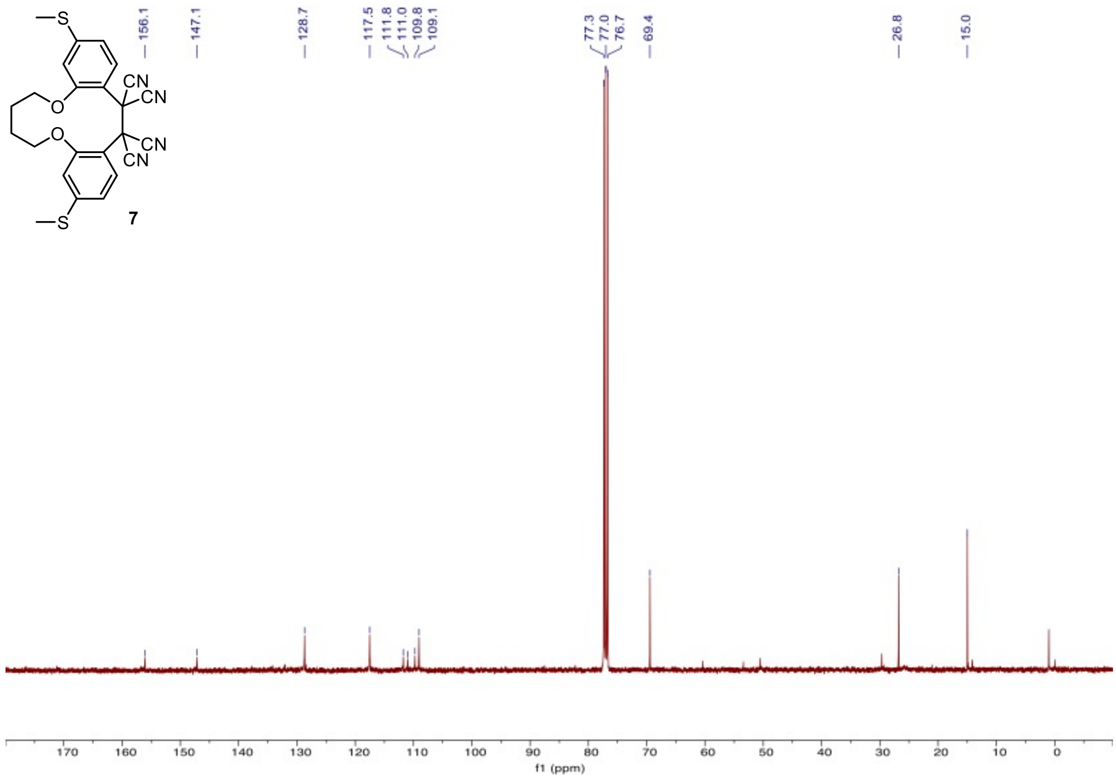

Figure S24. ^{13}C NMR spectrum of compound **7** in $CDCl_3$.

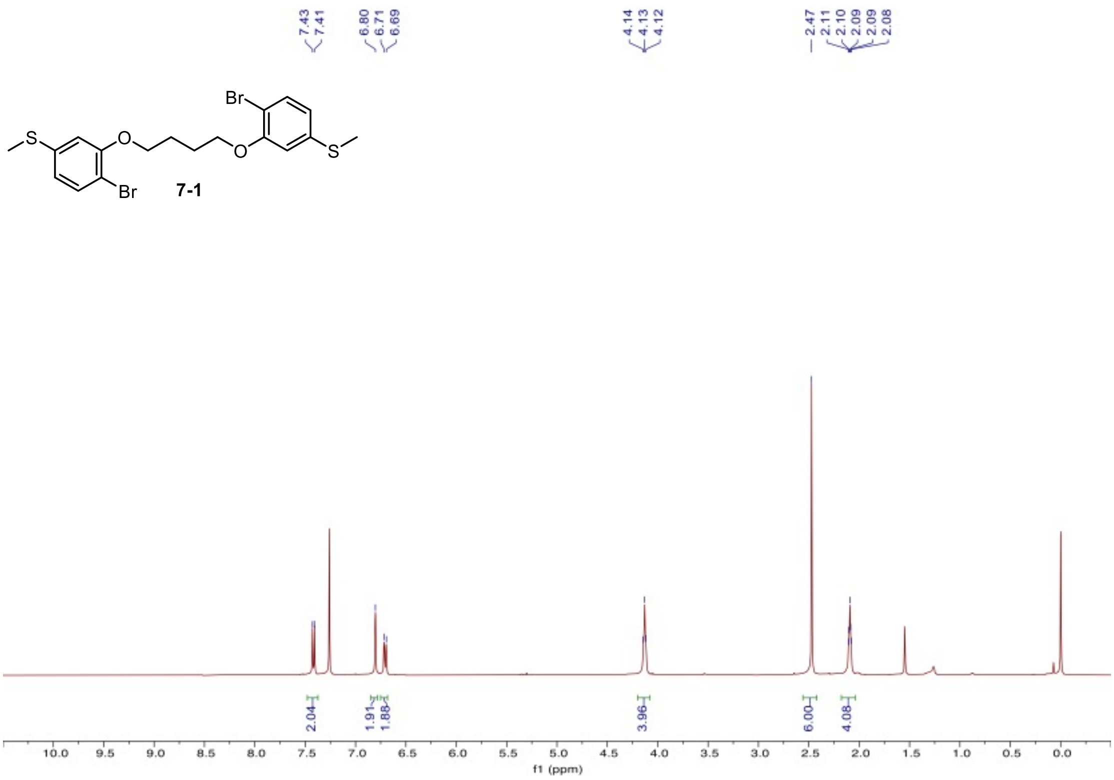

Figure S25. ^{1}H NMR spectrum of compound **7-1** in $CDCl_3$.

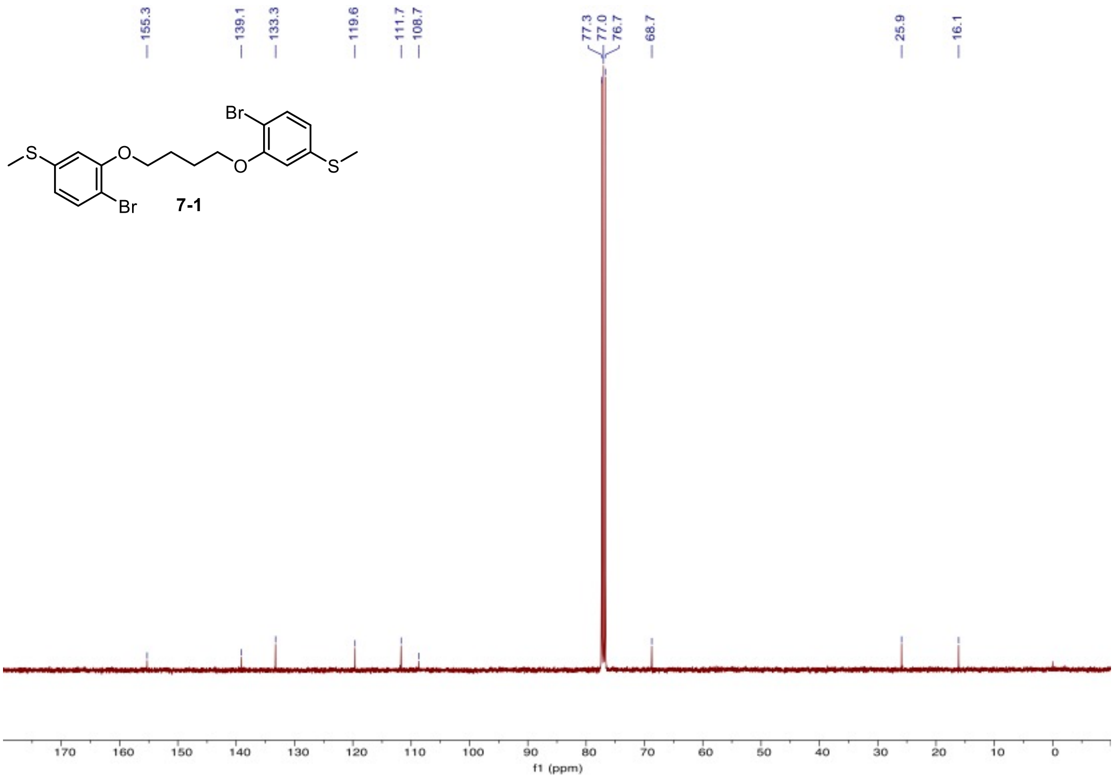

Figure S26. ^{13}C NMR spectrum of compound **7-1** in $CDCl_3$.

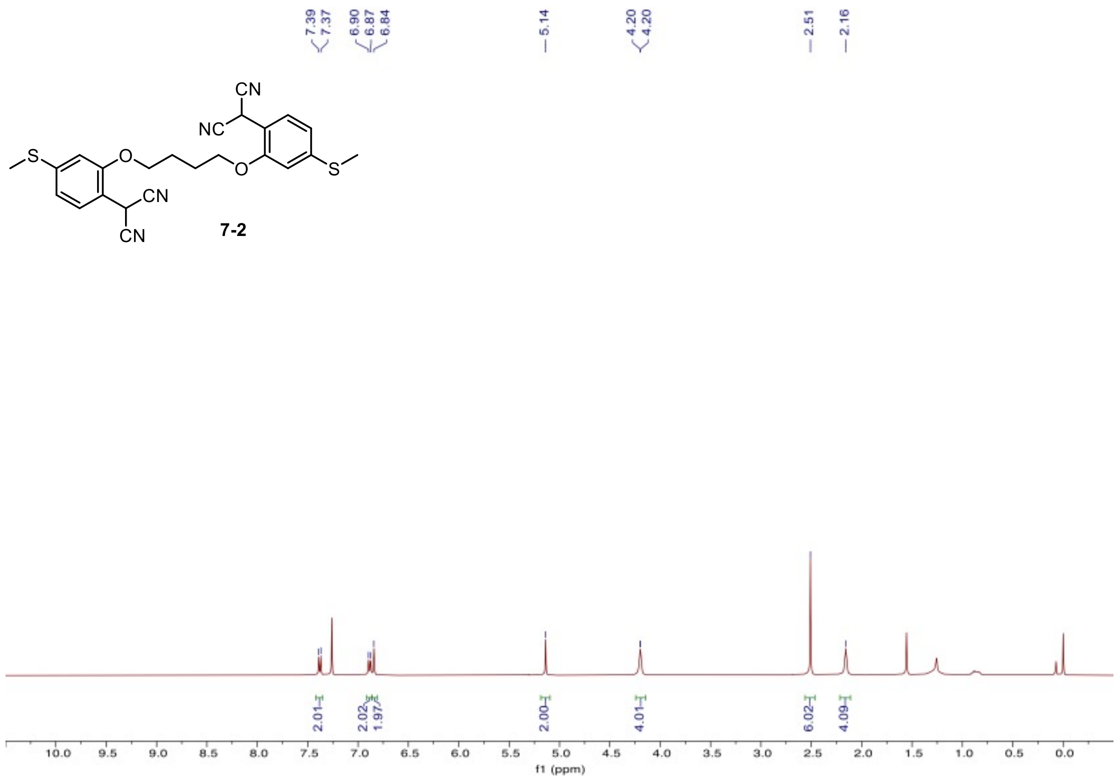

Figure S27. ^{1}H NMR spectrum of compound **7-2** in $CDCl_3$.

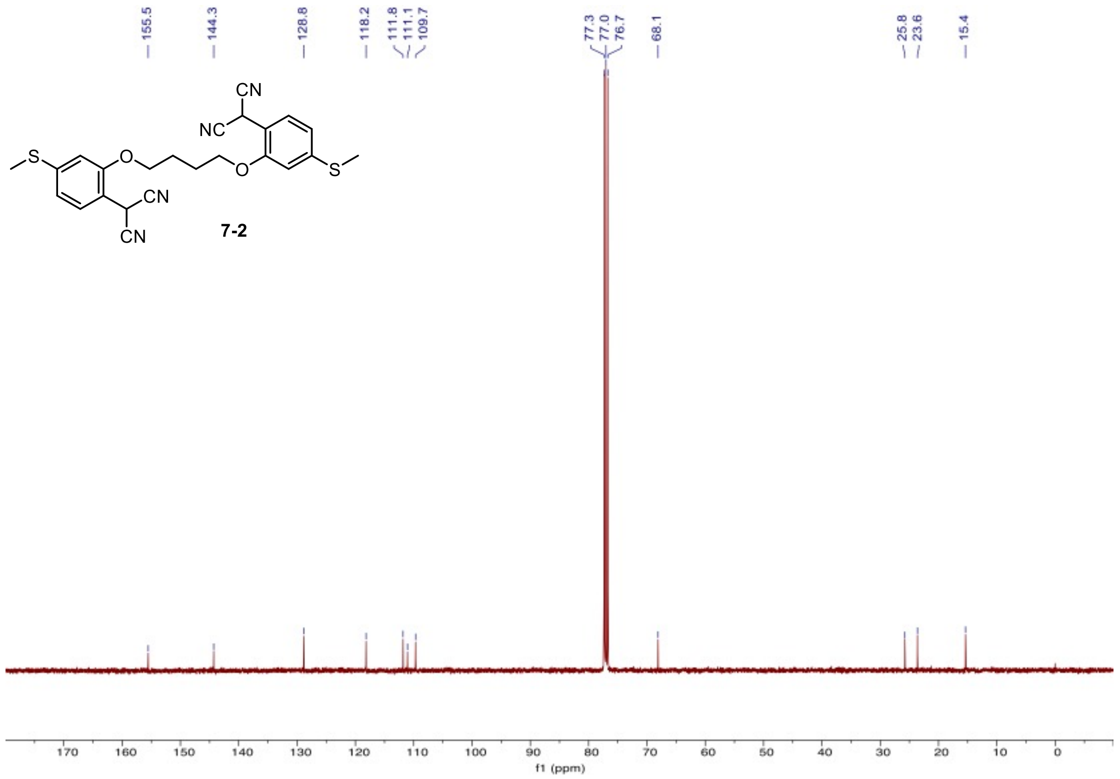

Figure S28. ^{13}C NMR spectrum of compound **7-2** in $CDCl_3$.

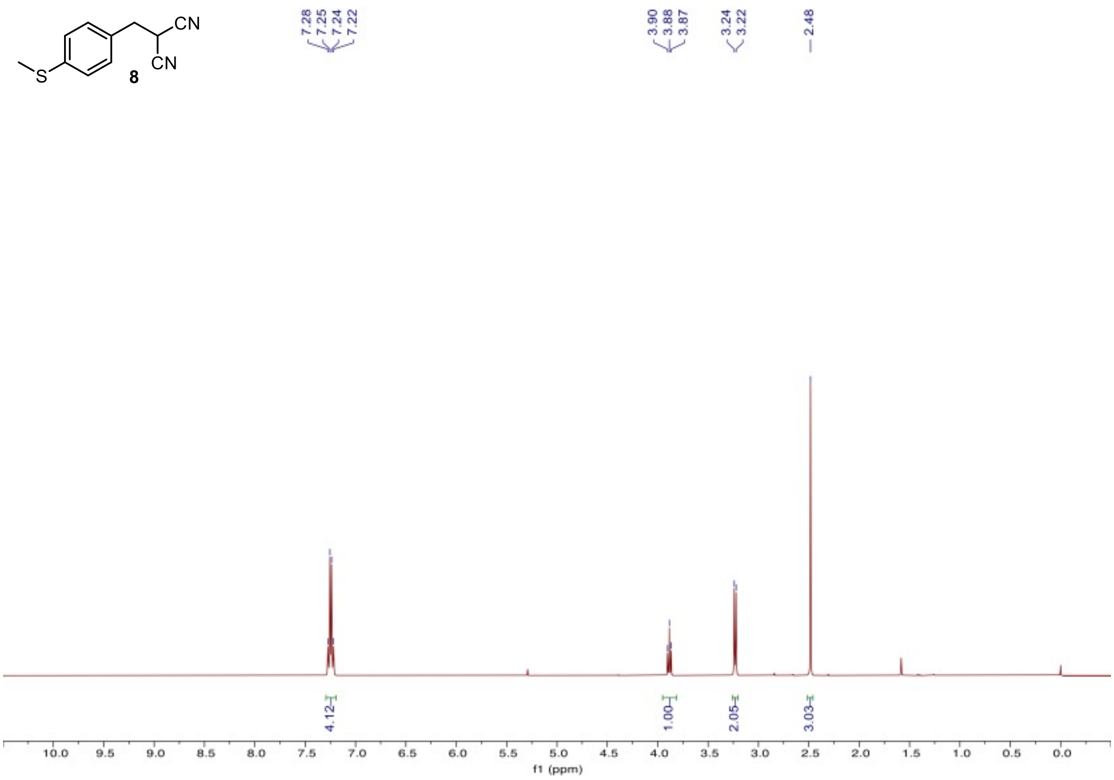

Figure S29. ^{1}H NMR spectrum of compound **8** in $CDCl_3$.

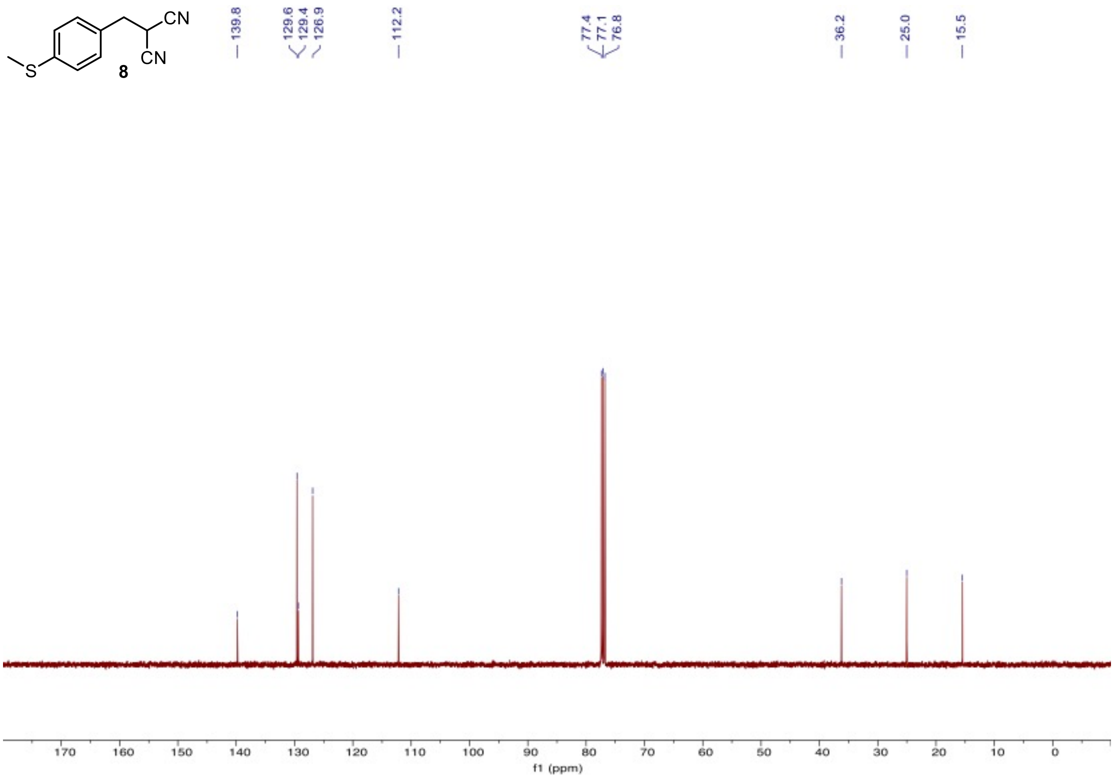

Figure S30. ^{13}C NMR spectrum of compound **8** in $CDCl_3$.

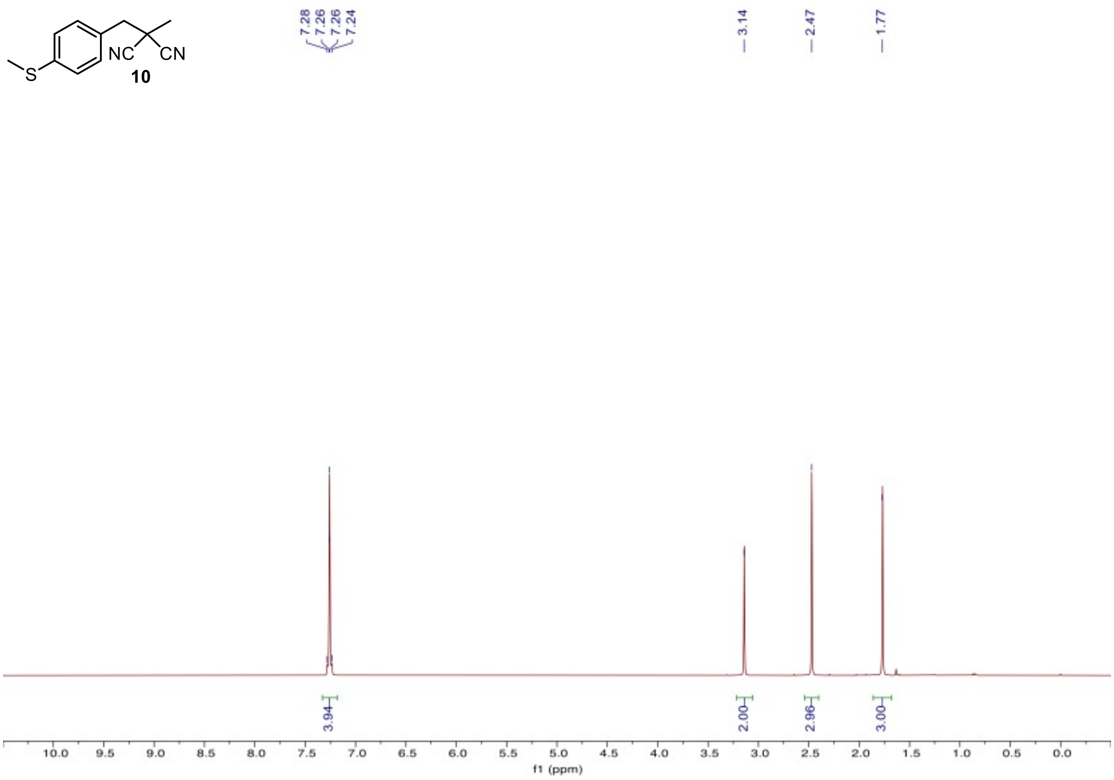

Figure S31. ^{1}H NMR spectrum of compound **10** in $CDCl_3$.

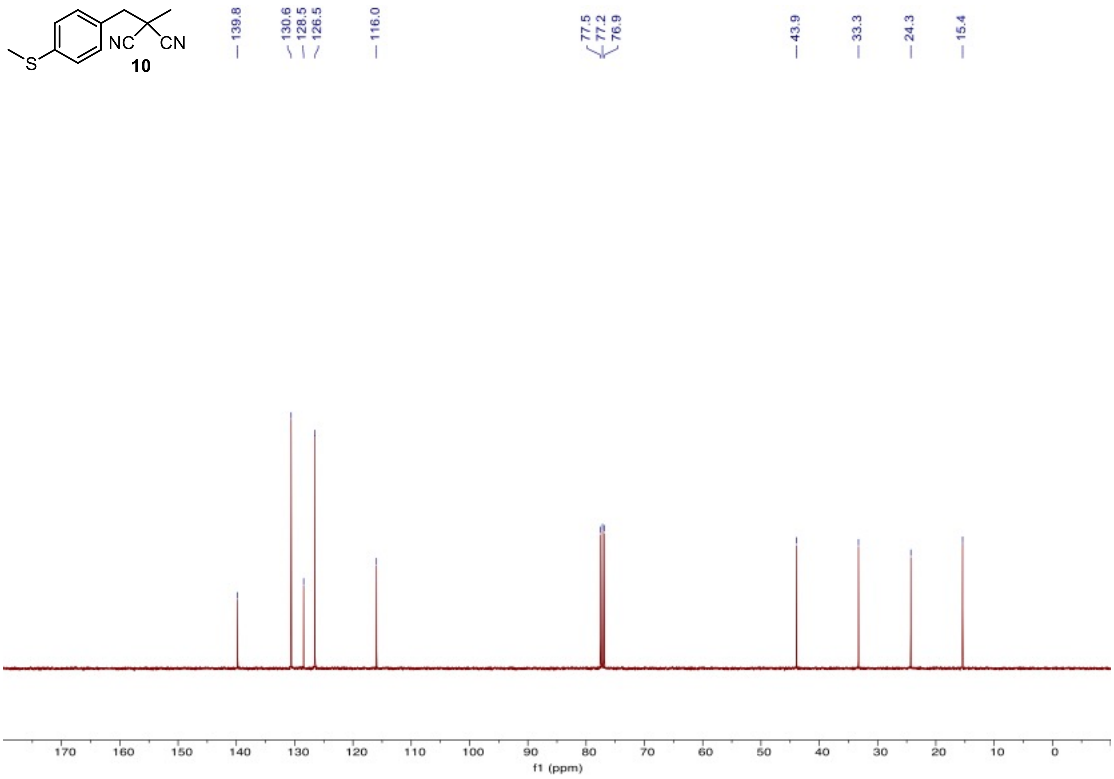

Figure S32. ^{13}C NMR spectrum of compound **10** in $CDCl_3$.

V. Details of the tight-binding model

V.1 Descriptions for the Hamiltonian

Tight-binding or Hückel model can be easily applied to give the Hamiltonian of the conjugated structures when calculating the transmission function of the molecular junctions.[3, 4] For studying 1highG and 3G, we treat each junction consisting of 8 separate hopping sites. The on-site energy of the C atoms in the phenyl ring backbone ϵ_B is set to 0. Hopping integral between nearest-neighbor C−C interaction sites is denoted by t, where $t = 1$. The coupling between the molecule and the left (L) and right (R) gold electrodes are described by the self-energy matrices Γ_L and Γ_R, respectively, with the parameters γ_L and γ_R within these matrices quantifying the coupling strength and signifying the interaction sites.

To model the covalent C−Au linkage on the left side, we introduce a gateway state with a σ-orbital.[5] We first treat the covalent bonded Au and C atoms as elements in the Hamiltonian with appropriate on-site energies labeled as ϵ_{Au} and ϵ, where ϵ_{Au} is coupled with the left electrode. We next use two distinct parameters to characterize the electron transport: s for Au−C covalent coupling and ts for C−C covalent coupling, each representing the orbital interaction strength along the backbone. For the dative SMe→Au linkage on the right side, we use a relatively small coefficient γ_R in the self-energy matrix Γ_R. Simultaneously, we extend this simple model for considering the effects of the −CN substitution groups. The substituents are treated as a perturbation to the on-site energy (ϵ for C1 or ϵ_B for C2−C7) of the attached carbons.

V.2 Hamiltonian for the studied junctions

The transmissions calculated using a tight-binding model shown in manuscript Figures 3f and 3g and in Supporting Information Figure S7 are configured with the following general parameters $\epsilon_{Au} = 0.9t$, $s = 0.7t$, $ts = 0.9t$, $\gamma_L = 2t$, $\gamma_R = 0.1t$ and variable parameter ϵ and ϵ_B. The 8 x 8 matrix that represents the Hamiltonian for 1highG and 3G junctions is as follows:

$$H = \begin{bmatrix} \epsilon_{Au} & -s & 0 & 0 & 0 & 0 & 0 & 0 \\ -s & \epsilon & -ts & 0 & 0 & 0 & 0 & 0 \\ 0 & -ts & \epsilon_B & -t & 0 & 0 & 0 & -t \\ 0 & 0 & -t & \epsilon_B & -t & 0 & 0 & 0 \\ 0 & 0 & 0 & -t & \epsilon_B & -t & 0 & 0 \\ 0 & 0 & 0 & 0 & -t & \epsilon_B & -t & 0 \\ 0 & 0 & 0 & 0 & 0 & -t & \epsilon_B & -t \\ 0 & 0 & -t & 0 & 0 & 0 & -t & \epsilon_B \end{bmatrix}$$

The coupling matrices for the left and right electrodes Γ_L and Γ_R can be expressed as follows:

$$\Gamma_L = \begin{bmatrix} \gamma_L & 0 & 0 & 0 & 0 & 0 & 0 & 0 \\ 0 & 0 & 0 & 0 & 0 & 0 & 0 & 0 \\ 0 & 0 & 0 & 0 & 0 & 0 & 0 & 0 \\ 0 & 0 & 0 & 0 & 0 & 0 & 0 & 0 \\ 0 & 0 & 0 & 0 & 0 & 0 & 0 & 0 \\ 0 & 0 & 0 & 0 & 0 & 0 & 0 & 0 \\ 0 & 0 & 0 & 0 & 0 & 0 & 0 & 0 \\ 0 & 0 & 0 & 0 & 0 & 0 & 0 & 0 \end{bmatrix}, \Gamma_R = \begin{bmatrix} 0 & 0 & 0 & 0 & 0 & 0 & 0 & 0 \\ 0 & 0 & 0 & 0 & 0 & 0 & 0 & 0 \\ 0 & 0 & 0 & 0 & 0 & 0 & 0 & 0 \\ 0 & 0 & 0 & 0 & 0 & 0 & 0 & 0 \\ 0 & 0 & 0 & 0 & 0 & 0 & 0 & 0 \\ 0 & 0 & 0 & 0 & 0 & \gamma_R & 0 & 0 \\ 0 & 0 & 0 & 0 & 0 & 0 & 0 & 0 \\ 0 & 0 & 0 & 0 & 0 & 0 & 0 & 0 \end{bmatrix}$$

V.3 Calculations of the transmission functions

We use the nonequilibrium Green's function (NEGF) to calculate the transmissions of the junctions:

$$G(E) = \left[EI - (\hat{H} + \Gamma_L + \Gamma_R)\right]^{-1}$$

where E is the energy of the electron and I is the identity matrix. The transmission function $T(E)$ is given by[6]

$$T(E) = Tr[\Gamma_L G \Gamma_R G^{\dagger}]$$

VI. References

(1) Zhang, R.; Peterson, J. P.; Fischer, L. J.; Ellern, A.; Winter, A. H. Effect of Structure on the Spin–Spin Interactions of Tethered Dicyanomethyl Diradicals. *J. Am. Chem. Soc.* **2018**, *140* (43), 14308-14313.
(2) Zeng, G.; Liu, J.; Shao, Y.; Zhang, F.; Chen, Z.; Lv, N.; Chen, J.; Li, R. Selective Synthesis of β-Ketonitriles via Catalytic Carbopalladation of Dinitriles. *The Journal of Organic Chemistry* **2021**, *86* (1), 861-867.
(3) Hückel, E. Quanstentheoretische beiträge zum benzolproblem: II. quantentheorie der induzierten polaritäten. *Z. Angew. Phys.* **1931**, *72* (5), 310-337.
(4) Hoffmann, R. An extended Hückel theory. I. hydrocarbons. *The Journal of Chemical Physics* **1963**, *39* (6), 1397-1412.
(5) Widawsky, J. R.; Chen, W.; Vázquez, H.; Kim, T.; Breslow, R.; Hybertsen, M. S.; Venkataraman, L. Length-Dependent Thermopower of Highly Conducting Au–C Bonded Single Molecule Junctions. *Nano Lett.* **2013**, *13* (6), 2889-2894.
(6) Datta, S. *Electronic transport in mesoscopic systems*; Cambridge university press, 1997.
